# Investigating the Efficacy of the Web-Based Common Elements Toolbox (COMET) Single-Session Interventions in Improving UK University Student Well-Being: Randomized Controlled Trial

**DOI:** 10.2196/58164

**Published:** 2025-01-31

**Authors:** Jeffrey Lambert, Maria Loades, Noah Marshall, Nina Higson-Sweeney, Stella Chan, Arif Mahmud, Victoria Pile, Ananya Maity, Helena Adam, Beatrice Sung, Melanie Luximon, Keren MacLennan, Clio Berry, Paul Chadwick

**Affiliations:** 1 Department for Health Universtiy of Bath Bath United Kingdom; 2 Department of Psychology University of Bath Bath United Kingdom; 3 School of Psychology and Clinical Language Sciences University of Reading Reading United Kingdom; 4 School of Education University of Roehampton London United Kingdom; 5 Department of Psychology Kings College London London United Kingdom; 6 Department of Psychology University of Durham Durham United Kingdom; 7 Brighton and Sussex Medical School Brighton United Kingdom

**Keywords:** Common Elements Toolbox, mental well-being, online interventions, single-session interventions, university students

## Abstract

**Background:**

Mental health problems in university students are associated with many negative outcomes, yet there is a gap between need and timely access to help. Single-session interventions (SSIs) are designed to be scalable and accessible, delivering core evidence-based intervention components within a one-off encounter.

**Objective:**

COMET (Common Elements Toolbox) is an online self-help SSI that includes behavioral activation, cognitive restructuring, gratitude, and self-compassion. COMET has previously been evaluated in India, Kenya, and the United States with promising results. This study tests the acceptability, appropriateness, perceived utility, and efficacy of COMET among UK university students during the peripandemic period.

**Methods:**

We conducted a randomized controlled trial evaluating the efficacy of COMET compared with a control group, with 2- and 4-week follow-ups. Outcome variables were subjective well-being, depression severity, anxiety severity, positive affect, negative affect, and perceived stress. We also measured intervention satisfaction immediately after completion of COMET. All UK university students with access to the internet were eligible to participate and were informed of the study online. The data were analyzed using linear mixed models and reported in accordance with the CONSORT-EHEALTH (Consolidated Standards of Reporting Trials of Electronic and Mobile Health Applications and Online Telehealth) checklist.

**Results:**

Of the 831 people screened, 468 participants were randomized to a condition, 407 completed the postintervention survey, 147 returned the 2-week follow-up survey, 118 returned the 4-week follow-up survey, and 89 returned both. Of the 239 randomized, 212 completed COMET. Significant between-group differences in favor of the COMET intervention were observed at 2-week follow-ups for subjective well-being (Warwick-Edinburgh Mental Well-Being Scale; mean difference [MD] 1.39, 95% CI 0.19-2.61; *P*=.03), depression severity (9-item Patient Health Questionnaire; MD –1.31, 95% CI –2.51 to –0.12; *P*=.03), and perceived stress (4-item Perceived Stress Scale; MD –1.33, 95% CI –2.10 to –0.57; *P*<.001). Overall, participants were satisfied with COMET, with the majority endorsing the intervention and its modules as acceptable, appropriate, and exhibiting high utility. The self-compassion module was most often reported as the participants’ favorite module and the behavioral activation module was their least favorite. Qualitative analysis revealed that participants found COMET generally accessible, but too long, and experienced immediate and long-term beneficial effects.

**Conclusions:**

This study demonstrated high engagement with the COMET intervention, along with preliminary short-term efficacy. Almost all participants completed the intervention, but study attrition was high. Participant feedback indicated a high level of overall satisfaction with the intervention, with perceived accessibility, immediate benefits, and potential long-term impact being notable findings. These findings support the potential value of COMET as a mental health intervention and highlight important areas for further improvement.

**Trial Registration:**

ClinicalTrials.gov NCT05718141; https://clinicaltrials.gov/ct2/show/NCT05718141

## Introduction

Mental health problems are common in young people, with at least one in five 16- to 24-year-olds being affected, particularly females [1,2]. University students represent a substantial portion of this population and are at high risk of developing mental health problems due to the unique stressors they may face, such as living away from home for the first time, financial hardship, balancing studying with other responsibilities, and changing social relationships [3,4]. A recent meta-analysis that investigated the mental health of university students found a pooled prevalence rate of 25% for depression and 14% for suicide-related outcomes (eg, suicidal ideation, suicide attempts) [5], with other studies finding that 35% of first-year students reported symptoms indicative of a lifetime mental health disorder [6]. For university students, mental health problems are associated with negative academic outcomes including lower grades [7] and increased dropout rates [8]. Even subthreshold depression and anxiety can lead to substantial impairment [9]. If left untreated, these mental health disorders can increase the risk of more severe mental health and physical health problems developing across the lifespan [10,11]. Early interventions are therefore vital in reducing disease burden on a societal level and are highlighted by students as a priority for mental health and well-being support [12].

The restrictions imposed during the COVID-19 pandemic further exacerbated the global mental health burden [13], particularly in young adults and university students [14,15], while also limiting access to informal and formal support systems. There is concerning evidence that only 20% of UK students struggling with their mental health during the pandemic sought help [16]. Even before the pandemic, only an average of 1 in 3 students experiencing psychological distress used mental health services [17]. Although universities in the United Kingdom do provide internal psychosocial support services for students, such as cognitive behavioral therapy (CBT) and counseling, there are numerous barriers to help-seeking, including self-reliance, poor mental health literacy, feeling too uncertain or unwell to seek help, lack of knowledge on how to access university services, preferences for alternative support (eg, online), and stigma [18-21]. Thus, there is a substantial gap between need and treatment access in this population. Even when support has been accessed, disengaging before completing the full course of treatment is common, meaning that some do not receive the full dose and optimal benefits [22].

Digital interventions (eg, online CBT) are one way of overcoming barriers students may experience in accessing mental health support by addressing concerns regarding anonymity, privacy, accessibility, and stigma [20]. Digital interventions also have a growing evidence base supporting their efficacy in addressing mental health problems [23,24]. For example, a recent network meta-analysis found that guided self-help interventions (including digital support) were more effective in reducing depression than waitlist control conditions [25]. Another meta-analysis, which compared 10 digital multisession interventions of a mean duration of 4 weeks with active and waitlist controls in university students, found small but significant improvements in psychological well-being after the intervention [26]. However, despite the promise of digital support, engagement is less than optimal due to factors such as lack of time and interest [27]. One way to address poor engagement with digital interventions is through online single-session interventions (SSIs). SSIs have the advantage of being more scalable and accessible because they are designed to deliver the core components of an active intervention within a one-off encounter, without an expectation that an individual will engage in longer-term therapy. Thus, SSIs could be a useful and effective addition to the suite of therapeutic options offered by university student services, which tend to be longer courses of treatment [28-34].

One such example is COMET (Common Elements Toolbox), an online (web-based) SSI without therapist contact. COMET was originally designed by Professor Rob de Rubeis’ team [35] for adolescents in India, and subsequently adapted and evaluated in US college students. COMET includes 4 modules based on evidence-based principles, namely, (1) behavioral activation (BA); (2) cognitive restructuring; (3) gratitude, from the discipline of positive psychology [36]; and (4) self-compassion. Versions of COMET have been developed with Kenyan and Indian adolescents and tested with US graduate students during the pandemic [35,37,38]. These versions are acceptable and useful, with US postgraduate students reporting pre- to post-program improvements in their perceived ability to manage the personal and psychological impacts of objective conditions or events (secondary control [39]). However, this online SSI has yet to be implemented and evaluated in a randomized controlled trial (RCT) among university students in the United Kingdom, with potential cultural differences meaning that existing findings cannot simply be extrapolated into the UK context. Additionally, UK higher education is typically shorter and less expensive than in the United States, and UK students apply for specific courses, whereas US students can switch majors during their university years.

We aimed to test the efficacy of COMET, an online mental health SSI, in undergraduate and postgraduate university students. Specifically, we sought to address the following questions:

- Compared with attention control, does COMET improve the mental health and well-being of university students at 2- and 4-week follow-ups?
- Do demographic variables (ie, age or gender) or clinical variables (ie, baseline depression severity, anxiety severity, mental health diagnoses, or treatment status) moderate the efficacy of COMET at 2- and 4-week follow-ups?
- How do participants perceive the acceptability and appropriateness of COMET, how do their utility ratings compare across the 4 COMET modules, and which modules do they prefer?

## Methods

### Trial Design

The study was a 2-arm, individually (1:1) randomized controlled trial design, comparing short-term (2- and 4-week) mental health outcomes of UK university students exposed to COMET (ie, the intervention group) or an attention-control questionnaire (ie, the control group). A nontherapeutic control was chosen to reflect the fact that SSIs are often provided as an alternative to receiving no support or being put on a waiting list for another intervention. The study was reported in accordance with CONSORT-EHEALTH (Consolidated Standards of Reporting Trials of Electronic and Mobile Health Applications and Online Telehealth; Multimedia Appendix 1) recommendations for the reporting of RCTs of eHealth interventions [40] and the TIDieR (Template for Intervention Description and Replication) recommendations on the reporting of behavior change interventions [41]. The trial was registered at ClinicalTrials.gov (NCT05718141) toward the end of data collection.

### Important Changes to Methods After Trial Commencement

We identified a Qualtrics error in January 2022, with participants who had completed the baseline survey not receiving the automatic 2- and 4-week follow-up surveys. This error was resolved in February 2022. We emailed a final follow-up survey link to the 36 participants who had completed the intervention before resolution and were outside the 4-week follow-up window by 1 week to 2 months. We also emailed survey links to the 45 participants who had completed the intervention but were still within the 2-week follow-up window and the 37 participants within the 4-week follow-up window. The follow-up data from the 36 participants who were 1 week to 2 months outside of the 4-week follow-up window were excluded from the main analyses, with these participants reflected in attrition rates.

### Participants

The study was set in the nonclinical, peripandemic context of UK universities and recruited currently registered undergraduate and postgraduate students with internet access. Those without internet access and those younger than 16 years were not eligible. The decision to offer the intervention more widely rather than just within clinical settings was to minimize barriers to access.

During the recruitment phase, potential participants were informed of the study through study adverts shared via social media platforms (eg, Facebook, Twitter, Instagram, TikTok), university-held mailing lists, and mailing lists/newsletters of charities and organizations with an interest in student mental health, such as Student Minds. The study was also advertised via psychology research participation schemes at the University of Bath and the University of Reading, which provide students with credits in exchange for taking part in research studies. It was also promoted on research recruitment websites such as MQ Mental Health Participate and Call for Participants. All adverts directed participants to a Qualtrics survey (Silver Lake Technology Management, L.L.C.) where they could learn more information about the study and take part.

Using power calculations based on the effect size of a previous iteration of COMET on the 9-item Patient Health Questionnaire (PHQ-9) [38], to detect a small effect of *d*=0.3, we required 378 participants to complete follow-up. A previous RCT of an SSI in adolescents had an attrition rate of around 28% at 3 months [32]. However, given our shorter follow-up rate of 4 weeks, we raised the recruitment target to 473 to allow for 20% attrition.

### Measures and Materials

All study documentation, including the information sheet, consent form, baseline assessment survey, experimental conditions, posttreatment survey, and follow-up surveys, was accessed through the Qualtrics platform. Brief demographic information was collected by self-report, including age in years, gender identity (female, male, or other), sexual orientation (heterosexual, bisexual, homosexual, other, or unlisted), and ethnicity (White or White British, Asian or Asian British, Black or Black British, or mixed). Participants were also asked about their mental health in relation to diagnoses, past and current experiences, and treatment status.

### Measures of Mental Health and Well-Being

For all participants, mental health and well-being were assessed at 3 time points, including a baseline assessment pretreatment, a 2-week follow-up, and a 4-week follow-up. Several dimensions of mental health and well-being were assessed, including (1) subjective well-being, (2) depression severity, (3) anxiety severity, (4) positive affect, (5) negative affect, and (6) perceived stress.

The Warwick-Edinburgh Mental Well-Being Scale (WEMWBS), a commonly used measure of well-being [42], was used in this study. The WEMWBS has 14 items that capture participants’ feelings and thoughts that best describe their experience over the previous 2 weeks using a scale from 1 (none of the time) to 5 (all of the time). The WEMWBS has robust psychometric properties [43]. The WEMWBS demonstrated good reliability, with a Cronbach α of 0.88 at baseline, 0.82 at the 2-week follow-up, and 0.83 at the 4-week follow-up.

The PHQ-9, a commonly used measure for depressive symptoms [44], was also used in this study. The 9 items of the questionnaire (α=.84) capture the frequency of depressive symptoms over the preceding 2 weeks using a scale from 0 to 3. A total score of 0-4 indicates no depression, 5-9 indicates mild depression, 10-14 indicates moderate depression, 15-19 indicates moderately severe depression, and 20-24 indicates severe depression. The PHQ-9 has a sensitivity and specificity of 88% for detecting clinical depression [45]. The PHQ-9 has demonstrated good reliability, with a Cronbach α of 0.84 at baseline, 0.89 at the 2-week follow-up, and 0.86 at the 4-week follow-up.

The 7-item General Anxiety Disorder (GAD-7) checklist is a commonly used measure for evaluating symptoms of anxiety [46]. The 7 items of the checklist (α=.87) capture the frequency of anxious symptoms over the preceding 2 weeks using a scale from 0 to 3. A total score of 0-4 indicates no anxiety, 5-9 indicates mild anxiety, 10-14 indicates moderate anxiety, and ≥15 indicates severe anxiety. The GAD-7 has a sensitivity and specificity of 89% and 82% respectively [46]. The GAD-7 demonstrated good reliability, with a Cronbach α of 0.87 at baseline, 0.85 at the 2-week follow-up, and 0.84 at the 4-week follow-up.

The Positive and Negative Affect Schedule (PANAS) is a commonly used measure of participants’ affective states [47]. This scale includes two 10-item subscales measuring positive affect and negative affect on a 5-point Likert scale (1=very slightly or not at all and 5=extremely). The PANAS has been shown to be reliable and valid [48]. The PANAS demonstrated good reliability for positive affect, with a Cronbach α of 0.88 at baseline, 0.90 at the 2-week follow-up, and 0.91 at the 4-week follow-up. The PANAS also demonstrated good reliability for negative affect, with a Cronbach α of 0.87 at baseline, 0.85 at the 2-week follow-up, and 0.87 at the 4-week follow-up.

The 4-item Perceived Stress Scale (PSS-4 [49]) is an abbreviated, 4-item scale designed to measure the extent of perceived stress in individuals’ lives over 4 weeks. However, to better align with our assessment intervals, this scale was adapted to specifically assess perceived stress over 2 weeks. The PSS-4 demonstrated good reliability, with a Cronbach α of 0.78 at baseline, 0.84 at the 2-week follow-up, and 0.80 at the 4-week follow-up.

### Measures of Intervention Satisfaction

Immediately after completing COMET, satisfaction with the intervention was assessed within a posttreatment survey using measures of (1) perceived appropriateness (perceived fit, relevance, or compatibility of treatment to address a particular issue or problem), (2) perceived acceptability (perception that a given treatment is agreeable, palatable, or satisfactory), (3) perceived utility, and (4) module preferences. These measures were added after trial registration because, as the study progressed, it became evident that the initial measures might not fully capture participants’ experiences with COMET. Therefore, we aimed to obtain a more detailed understanding of the intervention’s acceptability and align with best practice guidance, which emphasizes the importance of ongoing assessment and refinement in accurately evaluating complex interventions.

Intervention Appropriateness Measure is a 4-item measure assessing intervention appropriateness. Each item has a 5-point scale ranging from 1 (completely disagree) to 5 (completely agree) [47]. The Intervention Appropriateness Measure demonstrated good reliability, with a Cronbach α of 0.90.

Acceptability of Interventions Measure is a 4-item measure assessing intervention acceptability. Each item has a 5-point scale ranging from 1 (completely disagree) to 5 (completely agree) [47]. The Acceptability of Interventions Measure demonstrated good reliability, with a Cronbach α of 0.92.

Perceived utility is a bespoke measure where participants in the intervention group were asked to rate their feelings toward each module concerning helpfulness, engagement, and intention to apply intervention techniques in their daily lives. These questions were measured on a 7-point Likert scale from 1 (strongly disagree) to 7 (strongly agree), with 3 items per module. Across each of the 4 COMET modules, perceived utility also demonstrated good reliability, with Cronbach α values ranging from 0.90 to 0.94.

Participants indicated their preferences toward the 4 modules with the prompt questions asked: “Which exercise was your favorite?” and “Which exercise was your least favorite?.” All participants in the intervention group were also asked to complete a free textbox asking about their experiences with COMET.

### Procedure

Interested students were directed to a web-based Qualtrics survey including an information sheet, a consent form, a baseline assessment survey, descriptions of the experimental conditions, a posttreatment survey, and a debrief sheet. The information sheet explained the purposes of the research and the process of data collection and management. Following completion of the consent form, participants were directed to complete a baseline assessment survey measuring participants’ mental health and well-being. Participants were then randomly assigned to the intervention or control condition using the automated simple randomization tool embedded within Qualtrics. Thus, the research team was blind to treatment allocation. However, due to the intervention’s nature, the actual treatment assignment was not concealed from participants.

In addition to the preintervention measures, participants in the control group were also asked to complete additional questionnaires at baseline, which acted as an attention control for COMET.

### Intervention

#### Overview

Participants randomized to the intervention condition received and completed COMET, an online self-guided SSI. The intervention was accessible via any device which could connect to the internet, without any need to register or download software. It was based on the core principles of CBT, combined with principles from positive psychology. All 4 COMET modules were designed to be completed in a single session, taking 60-75 minutes. The modules featured short reading exercises, informational videos, and writing tasks.

#### Behavioral Activation

In this module, participants could identify and reflect on activities that were important to them, list activities they found enjoyable and meaningful, reflect on why these activities mattered to them, and schedule in time to perform these activities in the weeks ahead.

#### Cognitive Restructuring

In this module, participants were invited to identify and reframe negative beliefs. They were first asked to read about a hypothetical character who is adjusting to changes in their routine. Then, using the character’s story as an example, they were asked to try to identify negative beliefs that the character may have been experiencing and ways the character could reframe the belief. They could then apply this technique to a situation in their own life.

#### Gratitude

In this module, participants could reflect and write about 3 things they were grateful for. They were then asked to think and write about things they noticed around them that they enjoyed and were grateful for.

#### Self-Compassion

In this final module, participants were asked to write a self-compassion letter to themselves, expressing compassion toward themselves just as they would toward a friend or family member. They were also requested to create a few sentences that they would like to hear when feeling self-critical.

Further rationale for all 4 modules can be found in previous publications [35,38]. Participants could only access the intervention once. Outcome measures were collected immediately after they completed COMET.

### Attention Control

Participants allocated to the attention control group were asked to complete 5 additional measures, including a Symptom Importance Rating Questionnaire, the Chalder Fatigue Questionnaire [48], the Pittsburgh Sleep Quality Index [50], the Snaith-Hamilton Pleasure Scale [51], and the Fatigue Associated With Depression Scale [52]. These measures are not reported in this study. These were not completed by the intervention group nor reported as main outcomes on the RCT and will be reported elsewhere.

Immediately upon completion of these conditions, a posttreatment survey collected data related to their satisfaction with the COMET (only for the intervention group) and brief demographic information (for both groups). A debrief sheet signposted information about sources of mental health and well-being support. The initial intervention modules and assessment measures were designed to take participants approximately 60-75 minutes to complete.

Emails were sent out at 2 and 4 weeks after the intervention asking participants to complete follow-up assessment measures, which were designed to take approximately 10-15 minutes at each time point. All assessments took place online. Those who completed both follow-up assessments were able to opt into a prize draw for 1 of 16 £50 (US $63) Amazon vouchers. Additionally, those who completed the study as part of a student research participation scheme could earn course credits for taking part.

As a result of the pseudo-anonymous nature of the study, distress management was based on signposting, without direct or personal contact from the research team. It was the responsibility of the participants to decide whether they acted on this advice. If a participant scored >0 on the PHQ-9 item which asks about suicidal ideation (item 9), the participant saw an additional pop-up box suggesting that they may want to seek extra help, with a list of potential sources and contact details. They were reminded that the research team will not routinely monitor the answers to these questions. However, these participants were still included in the study.

### Ethical Considerations

All research was performed in accordance with relevant guidelines and regulations set by the Declaration of Helsinki. Ethical approval was granted by the University of Bath Psychology Research Ethics Committee (reference 21-212). Reciprocity was also granted by the University of Reading and Kings College London. All participants provided informed consent.

### Analysis Plan

All quantitative data were analyzed descriptively overall and by each arm. Continuous data were summarized using means and SDs. Categorical data were presented using frequencies and percentages. Data on feasibility (ie, recruitment, intervention engagement, and outcome completion rates) and acceptability (ie, intervention acceptability and appropriateness) were reported along with baseline characteristics in the 2 trial arms. All outcome data were analyzed using linear mixed models, adjusted for individual-level variation in baseline measures.

To address the first research question, intervention and control groups were compared based on complete case data at (1) the 2-week follow-up and (2) the 4-week follow-up using intention to treat (ITT). Between-group differences are presented as adjusted mean differences (MDs) and 95% CIs. Effect sizes were also calculated for the results of this study. For the second research question, exploratory moderation analyses were conducted to determine if baseline characteristics moderated the relationship between group assignment (intervention or control group) and each outcome measure. Age, anxiety, and depression were not recoded into categories. Gender, diagnoses, and current treatment were dummy-coded into binary values. To address the third research question, ratings of acceptability and perceived utility (at postintervention) were summarized using means and SDs. Sensitivity analysis using multiple imputations was conducted to assess the likely impact of missing data. Data were first analyzed using ITT (ie, all participants randomized who provided follow-up data). The data were then analyzed using ITT with all imputed data.

We analyzed the free textbox data using principles from inductive content analysis [53] in conjunction with Braun et al’s [54] method for analyzing qualitative data collected from online surveys. Specifically, 2 research team members (BS and ML) independently familiarized themselves with all the qualitative data by reading and re-reading the responses. Next, BS and ML independently focused on 15-20 responses and generated initial codes. These initial codes were then applied to the remaining free-text responses, utilizing constant comparison to determine whether additional codes were warranted for different meanings. After coding the data separately, BS and ML convened to compare their codes and discuss patterns in the responses. Through discussion and iterative review of the qualitative data, with supervisory input from M Loades, they refined and finalized the themes. Illustrative quotes were then selected to exemplify each theme.

## Results

### Participant Flow

**Figure 1:**
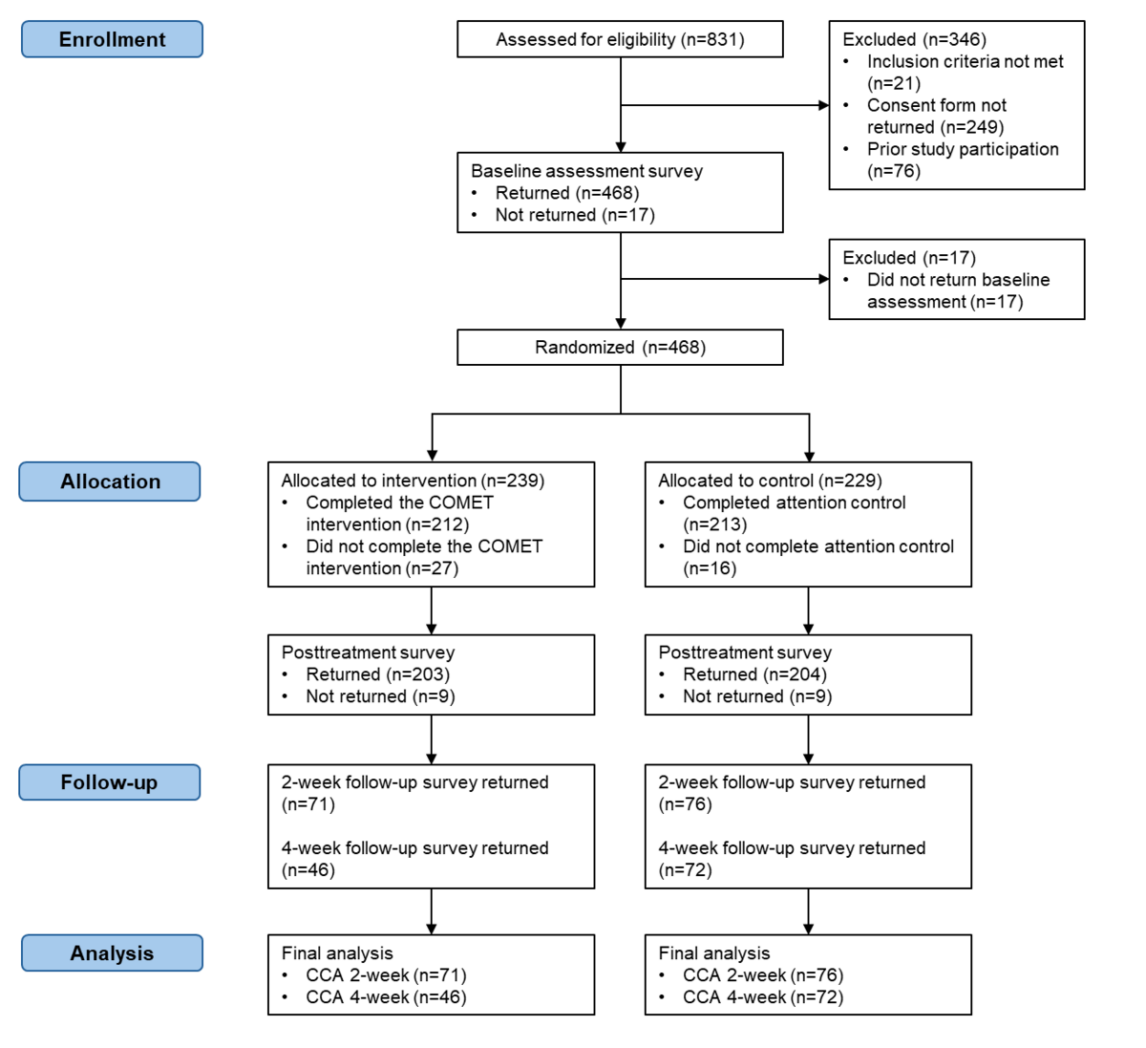
CONSORT (Consolidated Standards of Reporting Trials) flow diagram illustrating the progression of university students through the different phases of the randomized controlled trial. CCA: complete case analysis.

The participant flow is shown in Figure 1 (also see Multimedia Appendix 1). Participant recruitment and follow-up took place between September 2021 and December 2022. Of the 831 individuals assessed, 468 completed baseline measures and were randomized to either the COMET intervention (n=239) or the attention control (n=229). Of the 239 that were randomized and initiated COMET, 212 (88.7%) completed COMET, with 203 (84.9%) completing the posttreatment survey. A total of 213 out of 229 (93%) individuals completed the attention control, with 204 (89.1%) completing the postattention control survey. Of those randomized, 147 participants completed the 2-week follow-up survey, 118 completed the 4-week follow-up survey, and 89 participants completed both follow-up surveys.

### Baseline Data

The mean age of participants was 22.49 (SD 7.28) years, with most participants identifying as female (339/407, 83.3%), heterosexual (296/407, 72.7%), and White or White British (272/407, 66.8%); 126 of 407 (31%) participants had been previously diagnosed with a mental health disorder and 85 of 407 (20.9%) were actively receiving treatment for a mental health disorder during the study. Further, 162 of 407 (39.8%) participants had at least moderate depression and 95 of 407 (23.3%) had at least moderate anxiety (Table 1).

**Table 1:** Baseline demographic and clinical characteristics of university students participating in the randomized controlled trial, randomized to either the COMET^a^ intervention group or the waitlist control group.^b^

Characteristics			Intervention (n=203)		Control (n=204)		Total sample (n=407)
Age (years), mean (SD)			22.45 (7.13)		22.52 (7.44)		22.49 (7.28)
Gender (female), n (%)			172 (84.7)		167 (81.9)		339 (83.3)
**Sexual orientation, n (%)**							
	Heterosexual	143 (70.4)		153 (75.0)		296 (72.7)	
	Bisexual	29 (14.3)		27 (13.2)		56 (13.8)	
	Homosexual	7 (3.4)		5 (2.5)		12 (2.9)	
	Other or unlisted	24 (11.8)		19 (9.3)		43 (10.6)	
**Ethnicity, n (%)**							
	White or White British	137 (67.5)		135 (66.2)		272 (66.8)	
	Asian or Asian British	12 (5.9)		13 (6.4)		25 (6.1)	
	Black or Black British	3 (1.5)		5 (2.5)		8 (2.0)	
	Mixed	51 (25.1)		51 (25.0)		102 (25.1)	
Mental health diagnosis, n (%)			66 (32.5)		60 (29.4)		126 (31.0)
Receiving treatment, n (%)			42 (20.7)		43 (21.1)		85 (20.9)
9-item Patient Health Questionnaire ≥10, n (%)			81 (39.9)		81 (39.7)		162 (39.8)
7-item General Anxiety Disorder Checklist ≥10, n (%)			41 (20.2)		54 (26.5)		95 (23.3)

^a^COMET: Common Elements Toolbox.

^b^Demographic information was reported by participants after exposure to their allocated treatment condition. Completed posttreatment surveys were returned by 407 participants across the intervention (n=203) and control (n=204) conditions.

### Intervention Outcomes: Primary Analysis

At the 2-week follow-up, the complete-case ITT analysis showed that subjective well-being (WEMWBS) was significantly higher in the intervention group compared with the control group, with an MD of 1.39 (95% CI 0.19-2.61, *P*=.03) and a small effect size (Cohen *d*=0.24). Depression scores were also significantly lower in the intervention group compared with the control group (PHQ-9: MD –1.31, 95% CI –2.51 to –0.12; *P*=.03), with a small effect size (*d*=0.23). Perceived stress was also significantly lower in the intervention group compared with the control group (PSS-4: MD –1.33, 95% CI –2.10 to –0.57; *P*<.001), with a small effect size (*d*=0.37). No significant differences between groups in subjective well-being (*P*=.16), depression (*P*=.08), or perceived stress (*P*=.19) were observed at the 4-week follow-up. For the remaining outcome measures (ie, GAD-7, Negative Affect Scale, and Positive Affect Scale), no between-group differences were observed at the 2- (*P*=.31, *P*=.80, and *P*=.20) or 4-week (*P*=.33, *P*=.37, and *P*=.41) follow-ups (Table 2).

**Table 2:** Between-group differences in primary and secondary mental health outcomes among university students participating in the randomized controlled trial at the baseline, 2-week, and 4-week follow-up assessments^a^.

Outcome		Intervention, mean (SD)	Control, mean (SD)	Between-group difference (95% CI)	*P* value
**Subjective well-being (Warwick-Edinburgh Mental Well-Being Scale)**					
	Baseline	22.04 (4.85)	21.53 (5.17)	N/A^b^	N/A
	2 weeks	22.83 (4.37)	21.72 (4.76)	1.39 (0.19 to 2.61)	.03
	4 weeks	22.89 (4.39)	21.75 (5.06)	1.01 (–0.39 to 2.42)	.16
**Depression severity (** **9-item Patient Health Questionnaire** **)**					
	Baseline	8.30 (5.80)	9.05 (5.76)	N/A	N/A
	2 weeks	7.42 (5.27)	8.78 (6.41)	–1.31 (–2.51 to –0.12)	.03
	4 weeks	6.80 (5.12)	8.92 (6.34)	–1.39 (–2.92 to 0.15)	.08
**Anxiety severity (** **7-item General Anxiety Disorder Checklist** **)**					
	Baseline	6.05 (4.43)	6.73 (4.62)	N/A	N/A
	2 weeks	5.44 (3.59)	6.68 (4.80)	–0.51 (–1.49 to 0.47)	.31
	4 weeks	4.85 (3.33)	6.40 (4.63)	–0.59 (–1.81 to 0.61)	.33
**Negative affect**					
	Baseline	22.55 (7.33)	23.20 (8.02)	N/A	N/A
	2 weeks	21.29 (6.27)	22.76 (8.29)	–0.24 (–2.08 to 1.60)	.80
	4 weeks	20.41 (6.77)	22.85 (8.06)	–1.08 (–3.42 to 1.27)	.37
**Positive affect**					
	Baseline	28.02 (8.06)	27.16 (7.71)	N/A	N/A
	2 weeks	28.73 (7.73)	26.53 (8.11)	1.33 (–0.70 to 3.36)	.20
	4 weeks	28.76 (8.55)	26.89 (7.98)	1.06 (–1.47 to 3.58)	.41
**Perceived stress (** **4-item Perceived Stress Scale** **)**					
	Baseline	7.67 (3.27)	7.67 (3.07)	N/A	N/A
	2 weeks	6.86 (3.20)	8.04 (3.13)	–1.33 (–2.10 to –0.57)	<.001
	4 weeks	6.54 (2.96)	7.42 (3.43)	–0.68 (–1.69 to 0.33)	.19

^a^Analysis includes complete case data for 2 (n=147) and 4 weeks (n=118).

^b^N/A: not applicable.

### Intervention Outcomes: Moderation Analysis

For those receiving COMET, individuals who were not currently receiving treatment showed greater improvements in subjective well-being at both 2-week (B=–3.37, 95% CI –6.34 to –0.41; *P*=.03) and 4-week follow-ups (B=–4.11, 95% CI –7.57 to –0.65; *P*=.02) than those individuals who were receiving another treatment.

At the 4-week follow-up, younger individuals also showed greater improvements in depression (B=0.19, 95% CI 0.01-0.37; *P*=.04) and anxiety (B=0.15, 95% CI 0.01-0.30; *P*=.04) compared with their older counterparts.

Compared with those with lower baseline anxiety, individuals with higher baseline anxiety exhibited greater improvements in perceived stress at the 4-week follow-up (B=–0.23, 95% CI –0.45 to –0.01; *P*=.047).

No moderation effects were observed for gender, baseline depression severity, or mental health diagnoses. Please see Multimedia Appendix 2 for full analyses.

### Intervention Outcomes: Sensitivity Analyses

Missingness ranged from 0.00% to 68.42% for cases (mean 44.46%, SD 25.28%) and from 0.00% to 73.29% (mean 44.46%, SD 34.93%) for variables. Little's Missing Completely At Random (MCAR) Test was applied and indicated that the data were missing completely at random (*χ*^2^_100_=99.10, *P*=.51); hence, it was assumed that missingness was purely random and not related to any observed or unobserved data.

Given the high proportion of missing data, multiple imputation was carried out to estimate follow-up outcomes for all participants who did not provide data at the 2- and 4-week follow-ups (Multimedia Appendix 3). Depression and perceived stress were lower in the intervention group compared with the control at the 2-week follow-up but only bordering significance (PHQ-9: MD –0.53, 95% CI –1.10 to 0.04, *P*=.07; and PSS-4: MD=–0.57, 95% CI –1.15 to 0.01, *P*=.05). No between-group effects between the intervention and control were observed for any of the other variables or follow-up points.

### Intervention Acceptability

Overall, participants found COMET to be acceptable, with between 166 out of 203 (81.8%) and 188 out of 203 (92.6%) participants agreeing or strongly agreeing that they approved of, liked, and welcomed COMET and found it appealing. Participants also found COMET to be appropriate with between 162 out of 203 (79.8%) and 188 out of 203 (92.6%) agreeing or strongly agreeing that COMET was fitting, suitable, applicable, and a good match (Table 3). Each of COMET’s 4 modules was also perceived to have high utility (Table 4). Most participants at least slightly agreed that BA (169/203, 83.3%, to 181/203, 89.2%), cognitive restructuring (145/203, 71.4%, to 175/203, 86.2%), gratitude (174/203, 85.7%, to 182/203, 89.7%), and self-compassion (150/203, 73.9%, to 174/203, 85.7%) were helpful, engaging, and applicable. Participants most liked the self-compassion module (66/203, 32.5%) followed by gratitude (59/203, 29.1%), cognitive restructuring (55/203, 27.1%), and BA (23/203, 11.3%). Participants least liked the BA module (83/203, 40.9%), followed by self-compassion (51/203, 25.1%), gratitude (38/203, 18.7%), and cognitive restructuring (31/203, 15.3%).

**Table 3:** Assessment of the acceptability and appropriateness of the COMET^a^ intervention among university students (n=203).

Assessment		Agrees or strongly agrees, n (%)	Neutral, n (%)	Disagrees or strongly disagrees, n (%)
**Acceptability** **(** **Acceptability of Interventions Measure)**				
	Approve	188 (92.6)	12 (5.9)	3 (1.5)
	Appealing	166 (81.8)	27 (13.3)	10 (4.9)
	Like	177 (87.2)	21 (10.3)	5 (2.5)
	Welcome	177 (87.2)	23 (11.3)	3 (1.5)
**Appropriateness (Intervention Appropriateness Measure)**				
	Fitting	178 (87.7)	23 (11.3)	2 (1.0)
	Suitable	188 (92.6)	11 (5.4)	4 (2.0)
	Applicable	184 (90.6)	17 (8.4)	2 (1.0)
	Good match	162 (79.8)	33 (16.3)	8 (3.9)

^a^COMET: Common Elements Toolbox.

**Table 4:** Evaluation of the perceived utility of COMET’s^a^ 4 modules, focusing on whether university students found them helpful, engaging, and applicable to their personal or clinical needs (n=203).

Items of perceived utility by COMET module		Slightly agree, agree, strongly agree, n (%)	Neutral, n (%)	Slightly disagree, disagree, strongly disagree, n (%)
**Behavioral activation**				
	Helpful	181 (89.2)	9 (4.4)	13 (6.4)
	Engaging	178 (87.7)	12 (5.9)	13 (6.4)
	Applicable	169 (83.3)	15 (7.4)	19 (9.4)
**Cognitive restructuring**				
	Helpful	175 (86.2)	14 (6.9)	14 (6.9)
	Engaging	160 (78.8)	17 (8.4)	26 (12.8)
	Applicable	145 (71.4)	23 (11.3)	35 (17.2)
**Gratitude**				
	Helpful	182 (89.7)	9 (4.4)	12 (5.9)
	Engaging	177 (87.2)	12 (5.9)	14 (6.9)
	Applicable	174 (85.7)	9 (4.4)	20 (9.9)
**Self-compassion**				
	Helpful	174 (85.7)	17 (8.4)	12 (5.9)
	Engaging	170 (83.7)	18 (8.9)	15 (7.4)
	Applicable	150 (73.9)	26 (12.8)	27 (13.3)

^a^COMET: Common Elements Toolbox.

### Qualitative Analysis

The free-text content analysis of participants’ experiences of COMET resulted in the formulation of 4 categories: time/length, accessibility, immediate effects, and long-term effects. Participants expressed that COMET introduced valuable skills in a manner that was simple and easy to understand, with many participants also noting the immediate effects of increased positivity, relaxation, and self-compassion upon completion. They also reported that the intervention helped to improve their thinking and outlook by rationalizing worries and recognizing the positive aspects of their lives. Participants believed that COMET had the potential for long-term impact, especially in helping them plan positive activities to regain a healthy routine and restructure their thinking. However, participants did reflect that some of the exercises were too long or boring, which could have potentially contributed to study dropout. Several participants also reported technical issues (eg, images not loading), which acted as a potential barrier to engagement. See Multimedia Appendix 4 for full analysis.

## Discussion

### Principal Findings

UK university students engaged well with the COMET online SSI and exhibited small, significant improvements in well-being, depression severity, and perceived stress over a 2-week follow-up period compared with the control arm. Changes in anxiety severity, positive affect, and negative affect were nonsignificant. Exploratory analysis also revealed that COMET was potentially more effective at reducing stress for those with elevated symptoms of anxiety. We also found that COMET was largely well-received in terms of acceptability, appropriateness, and feasibility, although users commented that it was too long, and some had technical issues.

A high level of intervention completion was observed, with 212 of 239 (88.7%) students randomized to COMET completing the intervention. This rate is particularly noteworthy when compared with completion rates in multiweek digital interventions, where a significant drop-off in engagement is common. For example, a systematic review of digital mental health interventions for depression, anxiety, and well-being in students found that most studies saw high completion rates after module 1 but reduced engagement over time, with only a minority completing all modules [55]. The structure of multiweek interventions may unintentionally contribute to attrition, as participants might find it difficult to commit to long-term engagement due to competing demands, a lack of immediate benefits, or the gradual loss of motivation. By contrast, SSIs such as COMET potentially address this challenge by minimizing the time commitment required, making it easier for participants to complete the program in a single sitting.

While COMET led to improvements in well-being, depression, and perceived stress at the 2-week follow-up, these changes were not observed for anxiety or positive and negative affect and had disappeared by the 4-week follow-up. This contrasts with another SSI RCT in adolescents, which found sustained effects on depression at 3 months [32]. One possible reason for this difference is the inclusion criteria. The previous study included participants based on depression criteria, whereas COMET aimed to be more inclusive by not using such criteria. In COMET, the mean baseline PHQ-9 score was below 10 (the cutoff for moderate depression), and only 126 of 407 (31.0%) participants had any mental health diagnosis. This may have led to ceiling effects, limiting the potential for long-lasting changes. Moderation analysis further supports this, showing that the effects were more pronounced for those with elevated baseline symptoms and a previous diagnosis. Additionally, the analysis found that older participants benefited less from COMET than younger ones. This could reflect that during our recruitment period between September and December, older participants, likely in later years of study, may experience higher levels of depression and anxiety due to increased pressure related to final-year projects and concerns about future employment compared with first-year students [56].

Our observed effect sizes at week 2 for well-being (*d*=–0.24), depression (*d*=0.23), and perceived stress (*d*=0.37) compare favorably with other online SSIs [30,32,57]. However, after multiple imputations, the effect on well-being became nonsignificant, and the effects on depression and perceived stress were bordering on significance. These effect sizes are smaller than those of more extensive internet-based CBT programs for anxiety and depression, with a previous meta-analysis found to have pooled effects of *g*=0.51 (95% CI 0.29-0.73) in young adults [58]. However, SSIs such as COMET could make a considerable difference at the population level, given their scalability and potential reach [59]. It is particularly encouraging that participants mentioned in the free text feedback how they could apply their learning from COMET in their daily lives, aligning with student priorities for mental health support [12].

In terms of participant preference, self-compassion was found to be the most liked aspect of COMET, while BA received lower ratings. Several possible reasons were identified to explain this discrepancy. First, BA is typically designed to be delivered over multiple weeks [60,61], including goal setting and regular goal reviews. In the COMET intervention, which consisted of a single session, the absence of goal reviews may have undermined the efficacy of BA. Second, previous studies have found self-compassion to be particularly valuable for students adjusting to university life with its various demands and stressors [62]. Our qualitative findings also suggest that participants found self-compassion to be a helpful coping strategy in this population. Third, COMET had no inclusion criteria for elevated symptoms of depression or anxiety. BA primarily targets depression and may be most suitable for individuals experiencing depressive symptoms. The BASIL trial, for instance, found that participants without depression perceived the relevance of BA to be limited, although this was in older adults [63]. Fourth, while activity monitoring has previously been regarded as beneficial for students, such as during the transition to college [64], the contextual limitations imposed by the pandemic made BA less effective in supporting participants due to the restrictions on engaging in pleasurable activities.

Participant recruitment was challenging, and it took 15 months to recruit the current sample. Online SSIs aimed at adolescents in the United States have recruited considerably larger samples in much shorter periods via social media [32]. A likely explanation for the difference is the guaranteed vouchers as an incentive to participate [65], whereas in our study, they were entered into a prize draw. When recruiting university students with subclinical anxiety or depression symptoms, a study in the Netherlands found that emailing all students via the central student administration was the most effective strategy, as compared with flyers, media, and social media adverts, among others [66]. Similarly, campus-wide recruitment emails resulted in over 80% recruitment of the 651 participants in a US-based trial of a universal, web-based prevention program for anxiety and depression [67]. Unfortunately, we were unable to secure agreement within our institutions to do this in our study. The pandemic context within which we recruited also made it more difficult to recruit using flyers/posters in physical locations on campus, and we, therefore, relied predominantly on social media and undergraduate research participation schemes, although we did also list the study on the research studies section of the student services web page at a UK university.

Once students had signed up to participate, our study had considerable study attrition, like other studies of digital mental health interventions in student populations [68-72]. Our high attrition could be explained by the qualitative feedback provided, which indicated that some participants experienced COMET as too long, with others having technical issues. Other studies have found that students prefer interventions that they can use in short bursts of time [73], and it may be that future iterations of COMET could give choices so that students can choose which components they want to do. This would also add a degree of personalization, which young people say aids their engagement in online interventions [74]. Technical issues are a common barrier to engagement in digital mental health interventions [75].

### Strengths and Limitations

We evaluated an existing intervention in a novel population, using a broad, well-validated series of psychometric instruments which spanned different dimensions of mental health problems and well-being. This is important, given that no single measure captures all stakeholder priorities in university student mental health [76], and our comprehensive approach including both mental health symptom measures and a well-being measure allowed for a more holistic understanding of COMET’s impact.

Like other digital mental health intervention studies in university student samples [68-72], we experienced high attrition rates from the study. Although the absence of patterned missingness suggests that this attrition did not bias the results, it substantially impacted the sample size. While we recruited and randomized 468 participants, only 147 returned the 2-week follow-up survey (attrition: 321/468, 68.6%) and 118 returned the 4-week follow-up survey (attrition: 350/468, 74.8%). Our a priori calculations indicated that a sample size of 378 participants would be required to detect statistically significant differences in the PHQ-9. Therefore, we had insufficient power, and our findings may be prone to type II errors. After multiple imputations, many of our estimates became nonsignificant, except for depression and perceived stress at the 2-week follow-up, which were only marginally significant. This suggests that even with imputation, the effect size might have been smaller than initially thought, and the study may need a larger sample size to be sufficiently powered.

Most participants were young, White, heterosexual women, like other university mental health intervention studies [67,77]. While this helps to provide valuable insights into the effects of COMET within this demographic, it does pose a limitation regarding the generalizability of the findings to the broader spectrum of UK university students. Furthermore, due to insufficient diversity in the sample, exploratory moderation analyses for demographic variables could not be meaningfully conducted.

While the efficacy of COMET was established across various domains, it is important to note that contrary to real-world settings, participants were given either monetary rewards or course credit incentives for completing these interventions. This raises concerns about the applicability and genuine impact of COMET outside of an incentivized research context [78]. Accordingly, to ensure results are driven by inherent value and user commitment rather than external rewards, future research should examine the intervention’s impact in contexts devoid of external motivators.

This study also focused on short-term outcomes at 2- and 4-week intervals, leaving questions about sustained efficacy over the long term. This is particularly important for making fair comparisons with longer, multisession interventions that collect follow-up data over extended periods. Future investigations should emphasize extended follow-ups to provide a comprehensive understanding of an intervention’s enduring benefits [79]. In contemporary psychotherapeutic research, a benchmark of at least six months is considered standard.

Finally, we were unable to measure the amount of time participants spent on COMET due to limitations in the intervention platform’s functionality. It is possible that individuals who spent more time on the platform experienced greater benefits. Future research should investigate the total time spent on SSIs and examine whether time spent on different components varies among users.

### Future Directions

Future studies should further explore how best to support underserved groups (eg, explore the experiences of minority groups [80]). Artificial intelligence–driven adaptive trials may also help us to answer what works for whom [81]. To reach university students before mental health symptoms become functionally impairing, early interventions or prevention efforts may achieve greater reach by embedding them within courses and ensuring maximal engagement through coproduction [82].

### Conclusions

This study demonstrated the preliminary short-term efficacy of the COMET intervention, as evidenced by the significant between-group differences favoring the intervention at the 2-week follow-up. However, attrition was high, potentially biasing the results. Participant feedback indicated overall satisfaction with the intervention, with perceived accessibility, immediate benefits, and potential long-term impact being notable findings. These findings support the potential value of COMET as a mental health intervention and highlight important areas for further development in future SSI interventions.

## Appendix

Multimedia Appendix 1 CONSORT-EHEALTH (Consolidated Standards of Reporting Trials of Electronic and Mobile Health Applications and Online Telehealth) checklist.

Multimedia Appendix 2 Interaction between group allocation and age, gender, previous diagnosis, current treatment, baseline depression, and baseline anxiety.

Multimedia Appendix 3 Sensitivity analysis.

Multimedia Appendix 4 Qualitative analysis.

## Abbreviations

BA: behavioral activation
CBT: cognitive behavioral therapy
COMET: Common Elements Toolbox
CONSORT-EHEALTH: Consolidated Standards of Reporting Trials of Electronic and Mobile Health Applications and Online Telehealth
GAD-7: 7-item General Anxiety Disorder Checklist
IAM: Intervention Appropriateness Measure
ITT: intention to treat
MD: mean difference
PANAS: Positive and Negative Affect Schedule
PHQ-9: 9-item Patient Health Questionnaire
PSS-4: 4-item Perceived Stress Scale
RCT: randomized controlled trial
SSI: single-session intervention
TIDieR: Template for Intervention Description and Replication
WEMWBS: Warwick-Edinburgh Mental Well-Being Scale

## Data Availability

Data are available on reasonable request. The guarantor (M Loades) is willing to examine all requests for the deidentified dataset.
