# Supplementary material for: Investigating the Efficacy of the Web-Based Common Elements Toolbox (COMET) Single-Session Interventions in Improving UK University Student Well-Being: Randomized Controlled Trial
Source: J Med Internet Res. 2025 Jan 31;27:e58164. doi: 10.2196/58164 (PMC11829182; doi:10.2196/58164)
Supplement: Multimedia Appendix 1 [file jmir_v27i1e58164_app1.pdf]

CONSORT-EHEALTH (V 1.6.1) - Submission/Publication Form

The CONSORT-EHEALTH checklist is intended for authors of randomized trials evaluating web-based and Internet-based applications/interventions, including mobile interventions, electronic games (incl multiplayer games), social media, certain telehealth applications, and other interactive and/or networked electronic applications. Some of the items (e.g. all subitems under item 5 - description of the intervention) may also be applicable for other study designs.

The goal of the CONSORT EHEALTH checklist and guideline is to be
a) a guide for reporting for authors of RCTs,
b) to form a basis for appraisal of an ehealth trial (in terms of validity)

CONSORT-EHEALTH items/subitems are MANDATORY reporting items for studies published in the Journal of Medical Internet Research and other journals / scientific societies endorsing the checklist.

Items numbered 1., 2., 3., 4a., 4b etc are original CONSORT or CONSORT-NPT (non-pharmacologic treatment) items.
Items with Roman numerals (i., ii, iii, iv etc.) are CONSORT-EHEALTH extensions/clarifications.

As the CONSORT-EHEALTH checklist is still considered in a formative stage, we would ask that you also RATE ON A SCALE OF 1-5 how important/useful you feel each item is FOR THE PURPOSE OF THE CHECKLIST and reporting guideline (optional).

Mandatory reporting items are marked with a red *.
In the textboxes, either copy & paste the relevant sections from your manuscript into this form - please include any quotes from your manuscript in QUOTATION MARKS,
or answer directly by providing additional information not in the manuscript, or elaborating on why the item was not relevant for this study.

YOUR ANSWERS WILL BE PUBLISHED AS A SUPPLEMENTARY FILE TO YOUR PUBLICATION IN JMIR AND ARE CONSIDERED PART OF YOUR PUBLICATION (IF ACCEPTED).
Please fill in these questions diligently. Information will not be copyedited, so please use proper spelling and grammar, use correct capitalization, and avoid abbreviations.

DO NOT FORGET TO SAVE AS PDF _AND_ CLICK THE SUBMIT BUTTON SO YOUR ANSWERS ARE IN OUR DATABASE !!!

Citation Suggestion (if you append the pdf as Appendix we suggest to cite this paper in the caption):
Eysenbach G, CONSORT-EHEALTH Group
CONSORT-EHEALTH: Improving and Standardizing Evaluation Reports of Web-based and Mobile Health Interventions
J Med Internet Res 2011;13(4):e126

URL: http://www.jmir.org/2011/4/e126/
doi: 10.2196/jmir.1923
PMID: 22209829

jl2426@bath.ac.uk Switch accounts

Not shared

Draft not saved

* Indicates required question

Your name *

First Last

Dr Jeffrey Lambert

Primary Affiliation (short), City, Country *

University of Toronto, Toronto, Canada

University of Bath, Bath, UK

Your e-mail address *

abc@gmail.com

jl2426@bath.ac.uk

Title of your manuscript *

Provide the (draft) title of your manuscript.

Investigating the efficacy of the online Common Elements Toolbox (COMET) single session intervention in improving UK university student wellbeing: Randomized controlled trial

Name of your App/Software/Intervention *

If there is a short and a long/alternate name, write the short name first and add the long name in brackets.

COMET (Common Elements Toolbox)

Evaluated Version (if any)

e.g. "V1", "Release 2017-03-01", "Version 2.0.27913"

Your answer

Language(s) *

What language is the intervention/app in? If multiple languages are available, separate by comma (e.g. "English, French")

English

URL of your Intervention Website or App

e.g. a direct link to the mobile app on app in appstore (itunes, Google Play), or URL of the website. If the intervention is a DVD or hardware, you can also link to an Amazon page.

Your answer

URL of an image/screenshot (optional)

Your answer

Accessibility *

Can an enduser access the intervention presently?

- ( ) access is free and open
- ( ) access only for special usergroups, not open
- ( ) access is open to everyone, but requires payment/subscription/in-app purchases
- (x) app/intervention no longer accessible
- ( ) Other:

Primary Medical Indication/Disease/Condition *

e.g. "Stress", "Diabetes", or define the target group in brackets after the condition, e.g. "Autism (Parents of children with)", "Alzheimers (Informal Caregivers of)"

Mental health/wellbeing

Primary Outcomes measured in trial *

comma-separated list of primary outcomes reported in the trial

Subjective wellbeing

Secondary/other outcomes

Are there any other outcomes the intervention is expected to affect?

Depression severity, anxiety severity, positive affect, negative affect, and perceived stress

Recommended "Dose" *

What do the instructions for users say on how often the app should be used?

- ◯ Approximately Daily
- ◯ Approximately Weekly
- ◯ Approximately Monthly
- ◯ Approximately Yearly
- ◯ "as needed"
- ◉ Other: Once

Approx. Percentage of Users (starters) still using the app as recommended after 3 months *

- ◉ unknown / not evaluated
- ◯ 0-10%
- ◯ 11-20%
- ◯ 21-30%
- ◯ 31-40%
- ◯ 41-50%
- ◯ 51-60%
- ◯ 61-70%
- ◯ 71%-80%
- ◯ 81-90%
- ◯ 91-100%
- ◯ Other:

Overall, was the app/intervention effective? *

- ◯ yes: all primary outcomes were significantly better in intervention group vs control
- ◉ partly: SOME primary outcomes were significantly better in intervention group vs control
- ◯ no statistically significant difference between control and intervention
- ◯ potentially harmful: control was significantly better than intervention in one or more outcomes
- ◯ inconclusive: more research is needed
- ◯ Other:

Article Preparation Status/Stage *

At which stage in your article preparation are you currently (at the time you fill in this form)

- ◯ not submitted yet - in early draft status
- ◉ not submitted yet - in late draft status, just before submission
- ◯ submitted to a journal but not reviewed yet
- ◯ submitted to a journal and after receiving initial reviewer comments
- ◯ submitted to a journal and accepted, but not published yet
- ◯ published
- ◯ Other:

Journal *

If you already know where you will submit this paper (or if it is already submitted), please provide the journal name (if it is not JMIR, provide the journal name under "other")

- ○ not submitted yet / unclear where I will submit this
- ◉ Journal of Medical Internet Research (JMIR)
- ○ JMIR mHealth and UHealth
- ○ JMIR Serious Games
- ○ JMIR Mental Health
- ○ JMIR Public Health
- ○ JMIR Formative Research
- ○ Other JMIR sister journal
- ○ Other:

Is this a full powered effectiveness trial or a pilot/feasibility trial? *

- ○ Pilot/feasibility
- ◉ Fully powered

Manuscript tracking number *

If this is a JMIR submission, please provide the manuscript tracking number under "other" (The ms tracking number can be found in the submission acknowledgement email, or when you login as author in JMIR. If the paper is already published in JMIR, then the ms tracking number is the four-digit number at the end of the DOI, to be found at the bottom of each published article in JMIR)

- ◉ no ms number (yet) / not (yet) submitted to / published in JMIR
- ○ Other:

TITLE AND ABSTRACT

1a) TITLE: Identification as a randomized trial in the title

1a) Does your paper address CONSORT item 1a? *

I.e does the title contain the phrase "Randomized Controlled Trial"? (if not, explain the reason under "other")

- (selected) yes
- ( ) Other:

1a-i) Identify the mode of delivery in the title

Identify the mode of delivery. Preferably use “web-based” and/or “mobile” and/or “electronic game” in the title. Avoid ambiguous terms like “online”, “virtual”, “interactive”. Use “Internet-based” only if Intervention includes non-web-based Internet components (e.g. email), use “computer-based” or “electronic” only if offline products are used. Use “virtual” only in the context of “virtual reality” (3-D worlds). Use “online” only in the context of “online support groups”. Complement or substitute product names with broader terms for the class of products (such as “mobile” or “smart phone” instead of “iphone”), especially if the application runs on different platforms.

	1	2	3	4	5	
subitem not at all important	○	○	○	○	●	essential

Clear selection

Does your paper address subitem 1a-i? *

Copy and paste relevant sections from manuscript title (include quotes in quotation marks "like this" to indicate direct quotes from your manuscript), or elaborate on this item by providing additional information not in the ms, or briefly explain why the item is not applicable/relevant for your study

"Investigating the efficacy of the web-based Common Elements Toolbox"

1a-ii) Non-web-based components or important co-interventions in title

Mention non-web-based components or important co-interventions in title, if any (e.g., “with telephone support”).

	1	2	3	4	5	
subitem not at all important	(●)	( )	( )	( )	( )	essential

Clear selection

Does your paper address subitem 1a-ii?

Copy and paste relevant sections from manuscript title (include quotes in quotation marks "like this" to indicate direct quotes from your manuscript), or elaborate on this item by providing additional information not in the ms, or briefly explain why the item is not applicable/relevant for your study

"UK university student wellbeing"

1a-iii) Primary condition or target group in the title

Mention primary condition or target group in the title, if any (e.g., “for children with Type I Diabetes”) Example: A Web-based and Mobile Intervention with Telephone Support for Children with Type I Diabetes: Randomized Controlled Trial

	1	2	3	4	5	
subitem not at all important	( )	( )	(●)	( )	( )	essential

Clear selection

Does your paper address subitem 1a-iii? *

Copy and paste relevant sections from manuscript title (include quotes in quotation marks "like this" to indicate direct quotes from your manuscript), or elaborate on this item by providing additional information not in the ms, or briefly explain why the item is not applicable/relevant for your study

"UK university student wellbeing"

1b) ABSTRACT: Structured summary of trial design, methods, results, and conclusions

NPT extension: Description of experimental treatment, comparator, care providers, centers, and blinding status.

1b-i) Key features/functionalities/components of the intervention and comparator in the METHODS section of the ABSTRACT

Mention key features/functionalities/components of the intervention and comparator in the abstract. If possible, also mention theories and principles used for designing the site. Keep in mind the needs of systematic reviewers and indexers by including important synonyms. (Note: Only report in the abstract what the main paper is reporting. If this information is missing from the main body of text, consider adding it)

	1	2	3	4	5	
subitem not at all important	○	○	○	○	◉	essential

Clear selection

Does your paper address subitem 1b-i? *

Copy and paste relevant sections from the manuscript abstract (include quotes in quotation marks "like this" to indicate direct quotes from your manuscript), or elaborate on this item by providing additional information not in the ms, or briefly explain why the item is not applicable/relevant for your study

"an online self-help SSI, including behavioral activation, cognitive restructuring, gratitude, and self-compassion" "control group"

1b-ii) Level of human involvement in the METHODS section of the ABSTRACT

Clarify the level of human involvement in the abstract, e.g., use phrases like “fully automated” vs. “therapist/nurse/care provider/physician-assisted” (mention number and expertise of providers involved, if any). (Note: Only report in the abstract what the main paper is reporting. If this information is missing from the main body of text, consider adding it)

	1	2	3	4	5	
subitem not at all important	(●)	( )	( )	( )	( )	essential

Clear selection

Does your paper address subitem 1b-ii?

Copy and paste relevant sections from the manuscript abstract (include quotes in quotation marks "like this" to indicate direct quotes from your manuscript), or elaborate on this item by providing additional information not in the ms, or briefly explain why the item is not applicable/relevant for your study

Your answer

1b-iii) Open vs. closed, web-based (self-assessment) vs. face-to-face assessments in the METHODS section of the ABSTRACT

Mention how participants were recruited (online vs. offline), e.g., from an open access website or from a clinic or a closed online user group (closed usergroup trial), and clarify if this was a purely web-based trial, or there were face-to-face components (as part of the intervention or for assessment). Clearly say if outcomes were self-assessed through questionnaires (as common in web-based trials). Note: In traditional offline trials, an open trial (open-label trial) is a type of clinical trial in which both the researchers and participants know which treatment is being administered. To avoid confusion, use “blinded” or “unblinded” to indicated the level of blinding instead of “open”, as “open” in web-based trials usually refers to “open access” (i.e. participants can self-enrol). (Note: Only report in the abstract what the main paper is reporting. If this information is missing from the main body of text, consider adding it)

	1	2	3	4	5	
subitem not at all important	( )	( )	( )	( )	(●)	essential

Clear selection

Does your paper address subitem 1b-iii?

Copy and paste relevant sections from the manuscript abstract (include quotes in quotation marks "like this" to indicate direct quotes from your manuscript), or elaborate on this item by providing additional information not in the ms, or briefly explain why the item is not applicable/relevant for your study

"All UK university students with access to the internet were eligible to participate and were informed of the study online."

1b-iv) RESULTS section in abstract must contain use data

Report number of participants enrolled/assessed in each group, the use/uptake of the intervention (e.g., attrition/adherence metrics, use over time, number of logins etc.), in addition to primary/secondary outcomes. (Note: Only report in the abstract what the main paper is reporting. If this information is missing from the main body of text, consider adding it)

	1	2	3	4	5	
subitem not at all important	○	○	○	○	◉	essential

Clear selection

Does your paper address subitem 1b-iv?

Copy and paste relevant sections from the manuscript abstract (include quotes in quotation marks "like this" to indicate direct quotes from your manuscript), or elaborate on this item by providing additional information not in the ms, or briefly explain why the item is not applicable/relevant for your study

"Of the 239 randomized 212 completed COMET"

1b-v) CONCLUSIONS/DISCUSSION in abstract for negative trials

Conclusions/Discussions in abstract for negative trials: Discuss the primary outcome - if the trial is negative (primary outcome not changed), and the intervention was not used, discuss whether negative results are attributable to lack of uptake and discuss reasons. (Note: Only report in the abstract what the main paper is reporting. If this information is missing from the main body of text, consider adding it)

	1	2	3	4	5	
subitem not at all important	○	○	◉	○	○	essential

Clear selection

Does your paper address subitem 1b-v?

Copy and paste relevant sections from the manuscript abstract (include quotes in quotation marks "like this" to indicate direct quotes from your manuscript), or elaborate on this item by providing additional information not in the ms, or briefly explain why the item is not applicable/relevant for your study

Your answer

INTRODUCTION

2a) In INTRODUCTION: Scientific background and explanation of rationale

2a-i) Problem and the type of system/solution

Describe the problem and the type of system/solution that is object of the study: intended as stand-alone intervention vs. incorporated in broader health care program? Intended for a particular patient population? Goals of the intervention, e.g., being more cost-effective to other interventions, replace or complement other solutions? (Note: Details about the intervention are provided in “Methods” under 5)

	1	2	3	4	5	
subitem not at all important	○	○	○	○	◉	essential

Clear selection

Does your paper address subitem 2a-i? *

Copy and paste relevant sections from the manuscript (include quotes in quotation marks "like this" to indicate direct quotes from your manuscript), or elaborate on this item by providing additional information not in the ms, or briefly explain why the item is not applicable/relevant for your study

"Single session interventions have the advantage of being more scalable and accessible because they are designed to deliver the core component(s) of an active intervention within a one-off encounter, without an expectation that an individual will engage in longer-term therapy. Thus, SSIs could be a useful and effective addition to the suite of therapeutic options offered by university student services, which tend to be longer courses of treatment"

2a-ii) Scientific background, rationale: What is known about the (type of) system

Scientific background, rationale: What is known about the (type of) system that is the object of the study (be sure to discuss the use of similar systems for other conditions/diagnoses, if appropiate), motivation for the study, i.e. what are the reasons for and what is the context for this specific study, from which stakeholder viewpoint is the study performed, potential impact of findings [2]. Briefly justify the choice of the comparator.

	1	2	3	4	5	
subitem not at all important	○	○	○	○	◉	essential

Clear selection

Does your paper address subitem 2a-ii? *

Copy and paste relevant sections from the manuscript (include quotes in quotation marks "like this" to indicate direct quotes from your manuscript), or elaborate on this item by providing additional information not in the ms, or briefly explain why the item is not applicable/relevant for your study

"Versions of COMET have been developed with Kenyan and Indian adolescents and tested with graduate students in the United States (US) in the pandemic context [36-38]. These previous versions have been found to be acceptable and useful; postgraduate students in the US reported pre- to post-program improvements in their perceived ability to manage the personal and psychological impacts from objective conditions or events (secondary control; [39]), namely the pandemic, alongside improvements in the perceived negative impact of the pandemic on their quality of life."

2b) In INTRODUCTION: Specific objectives or hypotheses

Does your paper address CONSORT subitem 2b? *

Copy and paste relevant sections from the manuscript (include quotes in quotation marks "like this" to indicate direct quotes from your manuscript), or elaborate on this item by providing additional information not in the ms, or briefly explain why the item is not applicable/relevant for your study

"We aimed to test the effectiveness of COMET, an online mental health SSI in undergraduate and postgraduate university students. Specifically, we sought to address the following questions:
1) Compared to an attention control, does COMET improve the mental health and wellbeing of university students at 2-week and 4-week follow-ups?
2) Do demographic variables (i.e., age, gender) or clinical variables (i.e., baseline depression severity, anxiety severity, mental health diagnoses, or treatment status) moderate the efficacy of COMET at 2-week and 4-week follow-ups?
3) How do ratings of perceived acceptability, appropriateness, and utility compare across the four COMET modules?"

METHODS

3a) Description of trial design (such as parallel, factorial) including allocation ratio

Does your paper address CONSORT subitem 3a? *

Copy and paste relevant sections from the manuscript (include quotes in quotation marks "like this" to indicate direct quotes from your manuscript), or elaborate on this item by providing additional information not in the ms, or briefly explain why the item is not applicable/relevant for your study

"The study was a 2-arm, individually (1:1) randomized controlled trial (RCT) design"

3b) Important changes to methods after trial commencement (such as eligibility criteria), with reasons

Does your paper address CONSORT subitem 3b? *

Copy and paste relevant sections from the manuscript (include quotes in quotation marks "like this" to indicate direct quotes from your manuscript), or elaborate on this item by providing additional information not in the ms, or briefly explain why the item is not applicable/relevant for your study

"A Qualtrics error was identified in January 2022, with participants who had completed the baseline survey not receiving the follow-up surveys which were due to automatically send 2-weeks and 4-weeks post-intervention. This error was resolved in February 2022. A manual email with a final follow-up survey link was sent to the 36 participants who had completed the intervention prior to resolution and were outside of both follow-up windows. Manual emails with survey links were sent to the 45 participants who had completed the intervention but were still within the 2-week follow-up window, and the 37 participants who were within the 4-week follow-up window."

3b-i) Bug fixes, Downtimes, Content Changes

Bug fixes, Downtimes, Content Changes: ehealth systems are often dynamic systems. A description of changes to methods therefore also includes important changes made on the intervention or comparator during the trial (e.g., major bug fixes or changes in the functionality or content) (5-iii) and other “unexpected events” that may have influenced study design such as staff changes, system failures/downtimes, etc. [2].

	1	2	3	4	5	
subitem not at all important	○	○	○	◉	○	essential

Clear selection

Does your paper address subitem 3b-i?

Copy and paste relevant sections from the manuscript (include quotes in quotation marks "like this" to indicate direct quotes from your manuscript), or elaborate on this item by providing additional information not in the ms, or briefly explain why the item is not applicable/relevant for your study

"A Qualtrics error was identified in January 2022, with participants who had completed the baseline survey not receiving the follow-up surveys which were due to automatically send 2-weeks and 4-weeks post-intervention. This error was resolved in February 2022. A manual email with a final follow-up survey link was sent to the 36 participants who had completed the intervention prior to resolution and were outside of both follow-up windows. Manual emails with survey links were sent to the 45 participants who had completed the intervention but were still within the 2-week follow-up window, and the 37 participants who were within the 4-week follow-up window."

4a) Eligibility criteria for participants

Does your paper address CONSORT subitem 4a? *

Copy and paste relevant sections from the manuscript (include quotes in quotation marks "like this" to indicate direct quotes from your manuscript), or elaborate on this item by providing additional information not in the ms, or briefly explain why the item is not applicable/relevant for your study

"Eligibility criteria were as follows: (i) registered undergraduate or postgraduate student at a university in the UK; and (ii) able to access the internet. Those without internet access and those under 16 years of age were not eligible."

4a-i) Computer / Internet literacy

Computer / Internet literacy is often an implicit “de facto” eligibility criterion - this should be explicitly clarified.

	1	2	3	4	5	
subitem not at all important	○	◉	○	○	○	essential

Clear selection

Does your paper address subitem 4a-i?

Copy and paste relevant sections from the manuscript (include quotes in quotation marks "like this" to indicate direct quotes from your manuscript), or elaborate on this item by providing additional information not in the ms, or briefly explain why the item is not applicable/relevant for your study

Your answer

4a-ii) Open vs. closed, web-based vs. face-to-face assessments:

Open vs. closed, web-based vs. face-to-face assessments: Mention how participants were recruited (online vs. offline), e.g., from an open access website or from a clinic, and clarify if this was a purely web-based trial, or there were face-to-face components (as part of the intervention or for assessment), i.e., to what degree got the study team to know the participant. In online-only trials, clarify if participants were quasi-anonymous and whether having multiple identities was possible or whether technical or logistical measures (e.g., cookies, email confirmation, phone calls) were used to detect/prevent these.

	1	2	3	4	5	
subitem not at all important	○	○	○	○	◉	essential

Clear selection

Does your paper address subitem 4a-ii? *

Copy and paste relevant sections from the manuscript (include quotes in quotation marks "like this" to indicate direct quotes from your manuscript), or elaborate on this item by providing additional information not in the ms, or briefly explain why the item is not applicable/relevant for your study

"During the recruitment phase, potential participants were informed of the study through study adverts shared via social media platforms (e.g., Facebook, Twitter, Instagram, TikTok), university held mailing lists, and mailing lists/newsletters of charities and organizations with an interest in student mental health, like Student Minds. The study was also advertised via psychology research participation schemes at the University of Bath and the University of Reading, which provide students with credits in exchange for taking part in research studies. It was also promoted on research recruitment websites such as MQ Mental Health Participate and Call for Participants."

4a-iii) Information giving during recruitment

Information given during recruitment. Specify how participants were briefed for recruitment and in the informed consent procedures (e.g., publish the informed consent documentation as appendix, see also item X26), as this information may have an effect on user self-selection, user expectation and may also bias results.

	1	2	3	4	5	
subitem not at all important	○	○	○	○	◉	essential

Clear selection

Does your paper address subitem 4a-iii?

Copy and paste relevant sections from the manuscript (include quotes in quotation marks "like this" to indicate direct quotes from your manuscript), or elaborate on this item by providing additional information not in the ms, or briefly explain why the item is not applicable/relevant for your study

"All study documentation, including the information sheet, consent form, baseline assessment survey, experimental conditions, post-treatment survey, and follow-up surveys, were accessed through the -Qualtrics platform."

4b) Settings and locations where the data were collected

Does your paper address CONSORT subitem 4b? *

Copy and paste relevant sections from the manuscript (include quotes in quotation marks "like this" to indicate direct quotes from your manuscript), or elaborate on this item by providing additional information not in the ms, or briefly explain why the item is not applicable/relevant for your study

"All study documentation, including the information sheet, consent form, baseline assessment survey, experimental conditions, post-treatment survey, and follow-up surveys, were accessed through the -Qualtrics platform."

4b-i) Report if outcomes were (self-)assessed through online questionnaires

Clearly report if outcomes were (self-)assessed through online questionnaires (as common in web-based trials) or otherwise.

	1	2	3	4	5	
subitem not at all important	○	○	○	○	◉	essential

Clear selection

Does your paper address subitem 4b-i? *

Copy and paste relevant sections from the manuscript (include quotes in quotation marks "like this" to indicate direct quotes from your manuscript), or elaborate on this item by providing additional information not in the ms, or briefly explain why the item is not applicable/relevant for your study

"All study documentation, including the information sheet, consent form, baseline assessment survey, experimental conditions, post-treatment survey, and follow-up surveys, were accessed through the -Qualtrics platform."

4b-ii) Report how institutional affiliations are displayed

Report how institutional affiliations are displayed to potential participants [on ehealth media], as affiliations with prestigious hospitals or universities may affect volunteer rates, use, and reactions with regards to an intervention.(Not a required item – describe only if this may bias results)

	1	2	3	4	5	
subitem not at all important	◉	○	○	○	○	essential

Clear selection

Does your paper address subitem 4b-ii?

Copy and paste relevant sections from the manuscript (include quotes in quotation marks "like this" to indicate direct quotes from your manuscript), or elaborate on this item by providing additional information not in the ms, or briefly explain why the item is not applicable/relevant for your study

Your answer

5) The interventions for each group with sufficient details to allow replication, including how and when they were actually administered

5-i) Mention names, credential, affiliations of the developers, sponsors, and owners

Mention names, credential, affiliations of the developers, sponsors, and owners [6] (if authors/evaluators are owners or developer of the software, this needs to be declared in a "Conflict of interest" section or mentioned elsewhere in the manuscript).

	1	2	3	4	5	
subitem not at all important	○	○	○	○	◉	essential

Clear selection

Does your paper address subitem 5-i?

Copy and paste relevant sections from the manuscript (include quotes in quotation marks "like this" to indicate direct quotes from your manuscript), or elaborate on this item by providing additional information not in the ms, or briefly explain why the item is not applicable/relevant for your study

"Intervention

Participants randomized to the intervention condition received and completed COMET, an online self-guided SSI. The intervention was accessible via any device which could connect to the internet, without any need to register or download software. It was based on the core principles of CBT, combined with principles from positive psychology. Each of COMET's four modules included short reading exercises, informational videos, and writing tasks.

● Behavioral Activation: In this module, participants could identify and reflect on activities that were important to them, list activities they found enjoyable and meaningful, reflect on why these activities mattered to them, and schedule in time to perform these activities in the weeks ahead.

● Cognitive Restructuring: In this module, participants were invited to identify and reframe negative beliefs. They were first asked to read about a hypothetical character who is adjusting to changes in their routine. Then, using the character's story as an example, they were asked to try to identify negative beliefs that the character may have been experiencing and ways the character could reframe the belief. They could then apply this technique to a situation in their own life.

● Gratitude: In this module, participants could reflect and write about three things they were grateful for. They were then asked to think and write about things they noticed around them that they enjoyed and were grateful for.

● Self-Compassion: In this final module, participants were asked to write a self-compassion letter to themselves, expressing compassion towards themselves just as they would towards a friend or family member. They were also requested to create a few sentences that they would like to hear when feeling self-critical.

Further rationale for all four modules can be found in previous publications [37, 38].

Attention Control

Participants allocated to the attention control group were asked to complete five additional measures, including a Symptom Importance Rating Questionnaire, the Chalder Fatigue Questionnaire [48], the Pittsburgh Sleep Quality Index [49], the Snaith-Hamilton Pleasure Scale [50], and the Fatigue Associated with Depression Scale [51]. These measures are not reported in this study. These were not completed by the intervention group nor reported as main outcomes on the RCT and will be reported elsewhere."

5-ii) Describe the history/development process

Describe the history/development process of the application and previous formative evaluations (e.g., focus groups, usability testing), as these will have an impact on adoption/use rates and help with interpreting results.

	1	2	3	4	5	
subitem not at all important	○	○	○	○	◉	essential

Clear selection

Does your paper address subitem 5-ii?

Copy and paste relevant sections from the manuscript (include quotes in quotation marks "like this" to indicate direct quotes from your manuscript), or elaborate on this item by providing additional information not in the ms, or briefly explain why the item is not applicable/relevant for your study

"One such example is COMET (Common Elements Toolbox) is an online (web-based) SSI without therapist contact. COMET includes four modules based on evidence-based principles, namely (i) behavioral activation, (ii) cognitive restructuring, (iii) gratitude, from the discipline of positive psychology [35] and (iv) self-compassion. Versions of COMET have been developed with Kenyan and Indian adolescents and tested with graduate students in the United States (US) in the pandemic context [36-38]."

5-iii) Revisions and updating

Revisions and updating. Clearly mention the date and/or version number of the application/intervention (and comparator, if applicable) evaluated, or describe whether the intervention underwent major changes during the evaluation process, or whether the development and/or content was “frozen” during the trial. Describe dynamic components such as news feeds or changing content which may have an impact on the replicability of the intervention (for unexpected events see item 3b).

	1	2	3	4	5	
subitem not at all important	◉	○	○	○	○	essential

Clear selection

Does your paper address subitem 5-iii?

Copy and paste relevant sections from the manuscript (include quotes in quotation marks "like this" to indicate direct quotes from your manuscript), or elaborate on this item by providing additional information not in the ms, or briefly explain why the item is not applicable/relevant for your study

Your answer

5-iv) Quality assurance methods

Provide information on quality assurance methods to ensure accuracy and quality of information provided [1], if applicable.

	1	2	3	4	5	
subitem not at all important	(selected)	○	○	○	○	essential

Clear selection

Does your paper address subitem 5-iv?

Copy and paste relevant sections from the manuscript (include quotes in quotation marks "like this" to indicate direct quotes from your manuscript), or elaborate on this item by providing additional information not in the ms, or briefly explain why the item is not applicable/relevant for your study

Your answer

5-v) Ensure replicability by publishing the source code, and/or providing screenshots/screen-capture video, and/or providing flowcharts of the algorithms used

Ensure replicability by publishing the source code, and/or providing screenshots/screen-capture video, and/or providing flowcharts of the algorithms used. Replicability (i.e., other researchers should in principle be able to replicate the study) is a hallmark of scientific reporting.

	1	2	3	4	5	
subitem not at all important	◉	○	○	○	○	essential

Clear selection

Does your paper address subitem 5-v?

Copy and paste relevant sections from the manuscript (include quotes in quotation marks "like this" to indicate direct quotes from your manuscript), or elaborate on this item by providing additional information not in the ms, or briefly explain why the item is not applicable/relevant for your study

Your answer

5-vi) Digital preservation

Digital preservation: Provide the URL of the application, but as the intervention is likely to change or disappear over the course of the years; also make sure the intervention is archived (Internet Archive, webcitation.org, and/or publishing the source code or screenshots/videos alongside the article). As pages behind login screens cannot be archived, consider creating demo pages which are accessible without login.

	1	2	3	4	5	
subitem not at all important	◉	○	○	○	○	essential

Clear selection

Does your paper address subitem 5-vi?

Copy and paste relevant sections from the manuscript (include quotes in quotation marks "like this" to indicate direct quotes from your manuscript), or elaborate on this item by providing additional information not in the ms, or briefly explain why the item is not applicable/relevant for your study

Your answer

5-vii) Access

Access: Describe how participants accessed the application, in what setting/context, if they had to pay (or were paid) or not, whether they had to be a member of specific group. If known, describe how participants obtained “access to the platform and Internet” [1]. To ensure access for editors/reviewers/readers, consider to provide a “backdoor” login account or demo mode for reviewers/readers to explore the application (also important for archiving purposes, see vi).

	1	2	3	4	5	
subitem not at all important	○	○	○	○	◉	essential

Clear selection

Does your paper address subitem 5-vii? *

Copy and paste relevant sections from the manuscript (include quotes in quotation marks "like this" to indicate direct quotes from your manuscript), or elaborate on this item by providing additional information not in the ms, or briefly explain why the item is not applicable/relevant for your study

"During the recruitment phase, potential participants were informed of the study through study adverts shared via social media platforms (e.g., Facebook, Twitter, Instagram, TikTok), university held mailing lists, and mailing lists/newsletters of charities and organizations with an interest in student mental health, like Student Minds. The study was also advertised via psychology research participation schemes at the University of Bath and the University of Reading, which provide students with credits in exchange for taking part in research studies. It was also promoted on research recruitment websites such as MQ Mental Health Participate and Call for Participants."

5-viii) Mode of delivery, features/functionalities/components of the intervention and comparator, and the theoretical framework

Describe mode of delivery, features/functionalities/components of the intervention and comparator, and the theoretical framework [6] used to design them (instructional strategy [1], behaviour change techniques, persuasive features, etc., see e.g., [7, 8] for terminology). This includes an in-depth description of the content (including where it is coming from and who developed it) [1],” whether [and how] it is tailored to individual circumstances and allows users to track their progress and receive feedback” [6]. This also includes a description of communication delivery channels and – if computer-mediated communication is a component – whether communication was synchronous or asynchronous [6]. It also includes information on presentation strategies [1], including page design principles, average amount of text on pages, presence of hyperlinks to other resources, etc. [1].

	1	2	3	4	5	
subitem not at all important	○	○	○	○	◉	essential

Clear selection

Does your paper address subitem 5-viii? *

Copy and paste relevant sections from the manuscript (include quotes in quotation marks "like this" to indicate direct quotes from your manuscript), or elaborate on this item by providing additional information not in the ms, or briefly explain why the item is not applicable/relevant for your study

"Intervention
Participants randomized to the intervention condition received and completed COMET, an online self-guided SSI. The intervention was accessible via any device which could connect to the internet, without any need to register or download software. It was based on the core principles of CBT, combined with principles from positive psychology. Each of COMET's four modules included short reading exercises, informational videos, and writing tasks.
● Behavioral Activation: In this module, participants could identify and reflect on activities that were important to them, list activities they found enjoyable and meaningful, reflect on why these activities mattered to them, and schedule in time to perform these activities in the weeks ahead.
● Cognitive Restructuring: In this module, participants were invited to identify and reframe negative beliefs. They were first asked to read about a hypothetical character who is adjusting to changes in their routine. Then, using the character's story as an example, they were asked to try to identify negative beliefs that the character may have been experiencing and ways the character could reframe the belief. They could then apply this technique to a situation in their own life.
● Gratitude: In this module, participants could reflect and write about three things they were grateful for. They were then asked to think and write about things they noticed around them that they enjoyed and were grateful for.
● Self-Compassion: In this final module, participants were asked to write a self-compassion letter to themselves, expressing compassion towards themselves just as they would towards a friend or family member. They were also requested to create a few sentences that they would like to hear when feeling self-critical.
Further rationale for all four modules can be found in previous publications [37, 38]."

5-ix) Describe use parameters

Describe use parameters (e.g., intended "doses" and optimal timing for use). Clarify what instructions or recommendations were given to the user, e.g., regarding timing, frequency, heaviness of use, if any, or was the intervention used ad libitum.

	1	2	3	4	5	
subitem not at all important	○	○	○	○	◉	essential

Clear selection

Does your paper address subitem 5-ix?

Copy and paste relevant sections from the manuscript (include quotes in quotation marks "like this" to indicate direct quotes from your manuscript), or elaborate on this item by providing additional information not in the ms, or briefly explain why the item is not applicable/relevant for your study

"Intervention

Participants randomized to the intervention condition received and completed COMET, an online self-guided SSI. The intervention was accessible via any device which could connect to the internet, without any need to register or download software. It was based on the core principles of CBT, combined with principles from positive psychology. Each of COMET's four modules included short reading exercises, informational videos, and writing tasks.

- Behavioral Activation: In this module, participants could identify and reflect on activities that were important to them, list activities they found enjoyable and meaningful, reflect on why these activities mattered to them, and schedule in time to perform these activities in the weeks ahead.
- Cognitive Restructuring: In this module, participants were invited to identify and reframe negative beliefs. They were first asked to read about a hypothetical character who is adjusting to changes in their routine. Then, using the character's story as an example, they were asked to try to identify negative beliefs that the character may have been experiencing and ways the character could reframe the belief. They could then apply this technique to a situation in their own life.
- Gratitude: In this module, participants could reflect and write about three things they were grateful for. They were then asked to think and write about things they noticed around them that they enjoyed and were grateful for.
- Self-Compassion: In this final module, participants were asked to write a self-compassion letter to themselves, expressing compassion towards themselves just as they would towards a friend or family member. They were also requested to create a few sentences that they would like to hear when feeling self-critical.

Further rationale for all four modules can be found in previous publications [37, 38]."

5-x) Clarify the level of human involvement

Clarify the level of human involvement (care providers or health professionals, also technical assistance) in the e-intervention or as co-intervention (detail number and expertise of professionals involved, if any, as well as “type of assistance offered, the timing and frequency of the support, how it is initiated, and the medium by which the assistance is delivered”. It may be necessary to distinguish between the level of human involvement required for the trial, and the level of human involvement required for a routine application outside of a RCT setting (discuss under item 21 – generalizability).

	1	2	3	4	5	
subitem not at all important	○	○	○	○	●	essential

Clear selection

Does your paper address subitem 5-x?

Copy and paste relevant sections from the manuscript (include quotes in quotation marks "like this" to indicate direct quotes from your manuscript), or elaborate on this item by providing additional information not in the ms, or briefly explain why the item is not applicable/relevant for your study

"Participants randomized to the intervention condition received and completed COMET, an online self-guided SSI."

5-xi) Report any prompts/reminders used

Report any prompts/reminders used: Clarify if there were prompts (letters, emails, phone calls, SMS) to use the application, what triggered them, frequency etc. It may be necessary to distinguish between the level of prompts/reminders required for the trial, and the level of prompts/reminders for a routine application outside of a RCT setting (discuss under item 21 – generalizability).

	1	2	3	4	5	
subitem not at all important	●	○	○	○	○	essential

Clear selection

Does your paper address subitem 5-xi? *

Copy and paste relevant sections from the manuscript (include quotes in quotation marks "like this" to indicate direct quotes from your manuscript), or elaborate on this item by providing additional information not in the ms, or briefly explain why the item is not applicable/relevant for your study

"Participants randomized to the intervention condition received and completed COMET, an online self-guided SSI."

5-xii) Describe any co-interventions (incl. training/support)

Describe any co-interventions (incl. training/support): Clearly state any interventions that are provided in addition to the targeted eHealth intervention, as ehealth intervention may not be designed as stand-alone intervention. This includes training sessions and support [1]. It may be necessary to distinguish between the level of training required for the trial, and the level of training for a routine application outside of a RCT setting (discuss under item 21 – generalizability.

	1	2	3	4	5	
subitem not at all important	(•)	( )	( )	( )	( )	essential

Clear selection

Does your paper address subitem 5-xii? *

Copy and paste relevant sections from the manuscript (include quotes in quotation marks "like this" to indicate direct quotes from your manuscript), or elaborate on this item by providing additional information not in the ms, or briefly explain why the item is not applicable/relevant for your study

No co-interventions provided

6a) Completely defined pre-specified primary and secondary outcome measures, including how and when they were assessed

Does your paper address CONSORT subitem 6a? *

Copy and paste relevant sections from the manuscript (include quotes in quotation marks "like this" to indicate direct quotes from your manuscript), or elaborate on this item by providing additional information not in the ms, or briefly explain why the item is not applicable/relevant for your study

"For all participants, mental health and wellbeing was assessed at three timepoints, including: a baseline assessment pre-treatment, a 2-week follow-up, and a 4-week follow-up. Several dimensions of mental health and wellbeing were assessed, including: (i) subjective wellbeing, (ii) depression severity, (iii) anxiety severity, (iv) positive affect, (v) negative affect, and (vi) perceived stress.
Warwick-Edinburgh Mental Well-being Scale (WEMWBS), a commonly used measure of well-being [42]. The WEMWBS has 14 items (α = .88) that capture participants' feelings and thoughts that best describe their experience over the previous 2-weeks using a scale from 1 to 5. The WEMWBS has robust psychometric properties [43].
Patient Health Questionnaire-9 (PHQ-9), a commonly used measure for depressive symptoms [44]. The PHQ-9 has 9 items (α = .84) which capture the frequency of depressive symptoms over the preceding two weeks using a scale from 0-3. A total score of 0–4 indicates no depression, 5–9 indicates mild depression, 10–14 indicates moderate depression, 15– 19 indicates moderately severe depression and 20–24 indicates severe depression. The PHQ-9 has a sensitivity and specificity of 88% for detecting clinical depression [45].
General Anxiety Disorder 7-item Checklist (GAD-7), a commonly used measure for symptoms of anxiety [46]. The GAD-7 has 7 items (α = .87) which capture the frequency of anxious symptoms over the preceding two weeks using a scale from 0-3. A total score of 0–4 indicates no anxiety, 5–9 indicates mild anxiety, 10–14 indicates moderate anxiety and ≥15 indicates severe anxiety. The GAD-7 has a sensitivity and specificity of 89% and 82% respectively [46].
The Positive and Negative Affect Schedule (PANAS) is a commonly used measure of participants' affective states [47]. This scale includes two 10-item subscales measuring Positive Affect (PA) (α = .88) and Negative Affect (NA) (α = .87) on a 5-point Likert scale. The PANAS has been shown to be reliable and valid [48].
The Perceived Stress Scale-4 (Cohen et al., 1988) is an abbreviated, 4-item (α = .78) scale designed to measure the extent of perceived stress in individuals' lives over a 4-week period. However, to better align with our assessment intervals, this scale was adapted to specifically assess perceived stress over a 2-week period.
Measures of Intervention Satisfaction
Immediately after completing COMET, satisfaction with the intervention was assessed within a post-treatment survey using measures of (i) perceived appropriateness, (ii) perceived acceptability, (iii) perceived utility, and (iv) module preferences. Appropriateness Measure (IAM) and the Acceptability of Interventions Measure (AIM) [47]. Participants in the intervention group were asked to rate their feelings towards each module in terms of helpfulness, engagement, and intention to apply content going forward. These questions were measured on a 7-point Likert scale from 'Strongly disagree' to 'Strongly Agree', with three items per module. Participants indicated their preferences towards the four modules, with the prompt questions asked: 'Which exercise was your favorite?' and 'Which exercise was your least favorite?'. All participants in the intervention group were also asked to complete a free text box asking about their experiences of COMET."

6a-i) Online questionnaires: describe if they were validated for online use and apply CHERRIES items to describe how the questionnaires were designed/deployed

If outcomes were obtained through online questionnaires, describe if they were validated for online use and apply CHERRIES items to describe how the questionnaires were designed/deployed [9].

	1	2	3	4	5	
subitem not at all important	○	○	○	○	●	essential

Clear selection

Does your paper address subitem 6a-i?

Copy and paste relevant sections from manuscript text

"For all participants, mental health and wellbeing was assessed at three timepoints, including: a baseline assessment pre-treatment, a 2-week follow-up, and a 4-week follow-up. Several dimensions of mental health and wellbeing were assessed, including: (i) subjective wellbeing, (ii) depression severity, (iii) anxiety severity, (iv) positive affect, (v) negative affect, and (vi) perceived stress.
Warwick-Edinburgh Mental Well-being Scale (WEMWBS), a commonly used measure of well-being [42]. The WEMWBS has 14 items (α = .88) that capture participants' feelings and thoughts that best describe their experience over the previous 2-weeks using a scale from 1 to 5. The WEMWBS has robust psychometric properties [43].
Patient Health Questionnaire-9 (PHQ-9), a commonly used measure for depressive symptoms [44]. The PHQ-9 has 9 items (α = .84) which capture the frequency of depressive symptoms over the preceding two weeks using a scale from 0-3. A total score of 0–4 indicates no depression, 5–9 indicates mild depression, 10–14 indicates moderate depression, 15– 19 indicates moderately severe depression and 20–24 indicates severe depression. The PHQ-9 has a sensitivity and specificity of 88% for detecting clinical depression [45].
General Anxiety Disorder 7-item Checklist (GAD-7), a commonly used measure for symptoms of anxiety [46]. The GAD-7 has 7 items (α = .87) which capture the frequency of anxious symptoms over the preceding two weeks using a scale from 0-3. A total score of 0–4 indicates no anxiety, 5–9 indicates mild anxiety, 10–14 indicates moderate anxiety and ≥15 indicates severe anxiety. The GAD-7 has a sensitivity and specificity of 89% and 82% respectively [46].
The Positive and Negative Affect Schedule (PANAS) is a commonly used measure of participants' affective states [47]. This scale includes two 10-item subscales measuring Positive Affect (PA) (α = .88) and Negative Affect (NA) (α = .87) on a 5-point Likert scale. The PANAS has been shown to be reliable and valid [48].
The Perceived Stress Scale-4 (Cohen et al., 1988) is an abbreviated, 4-item (α = .78) scale designed to measure the extent of perceived stress in individuals' lives over a 4-week period. However, to better align with our assessment intervals, this scale was adapted to specifically assess perceived stress over a 2-week period.
Measures of Intervention Satisfaction
Immediately after completing COMET, satisfaction with the intervention was assessed within a post-treatment survey using measures of (i) perceived appropriateness, (ii) perceived acceptability, (iii) perceived utility, and (iv) module preferences. Appropriateness Measure (IAM) and the Acceptability of Interventions Measure (AIM) [47]. Participants in the intervention group were asked to rate their feelings towards each module in terms of helpfulness, engagement, and intention to apply content going forward. These questions were measured on a 7-point Likert scale from 'Strongly disagree' to 'Strongly Agree', with three items per module. Participants indicated their preferences towards the four modules, with the prompt questions asked: 'Which exercise was your favorite?' and 'Which exercise was your least favorite?'. All participants in the intervention group were also asked to complete a free text box asking about their experiences of COMET."

6a-ii) Describe whether and how “use” (including intensity of use/dosage) was defined/measured/monitored

Describe whether and how “use” (including intensity of use/dosage) was defined/measured/monitored (logins, logfile analysis, etc.). Use/adoption metrics are important process outcomes that should be reported in any ehealth trial.

	1	2	3	4	5	
subitem not at all important	◉	○	○	○	○	essential

Clear selection

Does your paper address subitem 6a-ii?

Copy and paste relevant sections from manuscript text

Your answer

6a-iii) Describe whether, how, and when qualitative feedback from participants was obtained

Describe whether, how, and when qualitative feedback from participants was obtained (e.g., through emails, feedback forms, interviews, focus groups).

	1	2	3	4	5	
subitem not at all important	○	○	◉	○	○	essential

Clear selection

Does your paper address subitem 6a-iii?

Copy and paste relevant sections from manuscript text

"All participants in the intervention group were also asked to complete a free text box asking about their experiences of COMET."

6b) Any changes to trial outcomes after the trial commenced, with reasons

Does your paper address CONSORT subitem 6b? *

Copy and paste relevant sections from the manuscript (include quotes in quotation marks "like this" to indicate direct quotes from your manuscript), or elaborate on this item by providing additional information not in the ms, or briefly explain why the item is not applicable/relevant for your study

"A Qualtrics error was identified in January 2022, with participants who had completed the baseline survey not receiving the follow-up surveys which were due to automatically send 2-weeks and 4-weeks post-intervention. This error was resolved in February 2022. A manual email with a final follow-up survey link was sent to the 36 participants who had completed the intervention prior to resolution and were outside of both follow-up windows. Manual emails with survey links were sent to the 45 participants who had completed the intervention but were still within the 2-week follow-up window, and the 37 participants who were within the 4-week follow-up window."

7a) How sample size was determined

NPT: When applicable, details of whether and how the clustering by care provides or centers was addressed

7a-i) Describe whether and how expected attrition was taken into account when calculating the sample size

Describe whether and how expected attrition was taken into account when calculating the sample size.

	1	2	3	4	5	
subitem not at all important	○	○	○	○	●	essential

Clear selection

Does your paper address subitem 7a-i?

Copy and paste relevant sections from manuscript title (include quotes in quotation marks "like this" to indicate direct quotes from your manuscript), or elaborate on this item by providing additional information not in the ms, or briefly explain why the item is not applicable/relevant for your study

"Using power calculations based on the effect size of a previous iteration of COMET on the PHQ-9 [38], to detect a small effect of d = 0.3, we required 378 participants to complete follow-up. A previous RCT of a SSI in adolescents had an attrition rate of around 28% at 3-months [32]. However, given our shorter follow-up rate of 4-weeks, we raised the recruitment target to 473 to allow for 20% attrition."

7b) When applicable, explanation of any interim analyses and stopping guidelines

Does your paper address CONSORT subitem 7b? *

Copy and paste relevant sections from the manuscript (include quotes in quotation marks "like this" to indicate direct quotes from your manuscript), or elaborate on this item by providing additional information not in the ms, or briefly explain why the item is not applicable/relevant for your study

"All quantitative data were analyzed descriptively overall and by each arm. Continuous data was summarized using means (M) and standard deviations (SD). Categorical data were presented using frequencies and percentages. Data on feasibility (i.e., recruitment, intervention engagement and outcome completion rates) and acceptability (i.e., intervention acceptability and appropriateness) was reported along with baseline characteristics in the two trial arms. All outcome data were analyzed using linear mixed models (LMMs), adjusted for individual-level variation in baseline measures.
To address the first research question, intervention and control groups were compared based on complete case data at (i) the 2-week follow-up, (ii) 4-week follow-up, and 2-week and 4-week follow-up data combined using intention to treat (ITT). Between-group differences are presented as adjusted mean differences and 95% confidence intervals (CIs). Effect sizes were also calculated for the results of this study. For the second research question, exploratory moderation analyses were conducted to determine if baseline characteristics moderated the relationship between group assignment (Intervention or Control group) and each outcome measure. To address the third research question, ratings of acceptability and perceived utility (at post-intervention) were summarized using means and standard deviations. Sensitivity analysis using multiple imputation was conducted to assess the likely impact of missing data. Data were first analyzed using intention to treat (ITT) (i.e., all participants randomized who provided follow-up data). The data was then analyzed using ITT with all imputed data.
Qualitative data generated in the free text boxes were analyzed using inductive content analysis, following the three phases of preparation, organizing, and reporting . Two members of the research team (BS, ML) completed the initial stages independently, and then collaborated at later stages to develop final categories. To begin, both team members immersed themselves in the data, by reading and re-reading the responses to make sense of what was going on. Then, they each focused on 15-20 randomly selected participant responses and generated initial open codes separately. These were then collated into coding sheets, which were used to code the rest of the responses using constant comparison. After doing this separately, the team members met to compare codes and ideas and begin grouping ideas into categories, which were then iterated through discussion and abstraction, with supervisory input from MEL. After finalizing and naming the categories, illustrative quotes were chosen to be included in the write-up."

8a) Method used to generate the random allocation sequence

NPT: When applicable, how care providers were allocated to each trial group

Does your paper address CONSORT subitem 8a? *

Copy and paste relevant sections from the manuscript (include quotes in quotation marks "like this" to indicate direct quotes from your manuscript), or elaborate on this item by providing additional information not in the ms, or briefly explain why the item is not applicable/relevant for your study

"Participants were then randomly assigned to the intervention or control condition using the automated simple randomization tool embedded within Qualtrics. Thus, the research team were blind to treatment allocation. However, due to the intervention's nature, actual treatment assignment was not concealed from participants."

8b) Type of randomisation; details of any restriction (such as blocking and block size)

Does your paper address CONSORT subitem 8b? *

Copy and paste relevant sections from the manuscript (include quotes in quotation marks "like this" to indicate direct quotes from your manuscript), or elaborate on this item by providing additional information not in the ms, or briefly explain why the item is not applicable/relevant for your study

"Participants were then randomly assigned to the intervention or control condition using the automated simple randomization tool embedded within Qualtrics. Thus, the research team were blind to treatment allocation. However, due to the intervention's nature, actual treatment assignment was not concealed from participants."

9) Mechanism used to implement the random allocation sequence (such as sequentially numbered containers), describing any steps taken to conceal the sequence until interventions were assigned

Does your paper address CONSORT subitem 9? *

Copy and paste relevant sections from the manuscript (include quotes in quotation marks "like this" to indicate direct quotes from your manuscript), or elaborate on this item by providing additional information not in the ms, or briefly explain why the item is not applicable/relevant for your study

"Participants were then randomly assigned to the intervention or control condition using the automated simple randomization tool embedded within Qualtrics. Thus, the research team were blind to treatment allocation. However, due to the intervention's nature, actual treatment assignment was not concealed from participants"

10) Who generated the random allocation sequence, who enrolled participants, and who assigned participants to interventions

Does your paper address CONSORT subitem 10? *

Copy and paste relevant sections from the manuscript (include quotes in quotation marks "like this" to indicate direct quotes from your manuscript), or elaborate on this item by providing additional information not in the ms, or briefly explain why the item is not applicable/relevant for your study

"Participants were then randomly assigned to the intervention or control condition using the automated simple randomization tool embedded within Qualtrics. Thus, the research team were blind to treatment allocation. However, due to the intervention's nature, actual treatment assignment was not concealed from participants."

11a) If done, who was blinded after assignment to interventions (for example, participants, care providers, those assessing outcomes) and how

NPT: Whether or not administering co-interventions were blinded to group assignment

11a-i) Specify who was blinded, and who wasn’t

Specify who was blinded, and who wasn’t. Usually, in web-based trials it is not possible to blind the participants [1, 3] (this should be clearly acknowledged), but it may be possible to blind outcome assessors, those doing data analysis or those administering co-interventions (if any).

	1	2	3	4	5	
subitem not at all important	○	○	○	○	◉	essential

Clear selection

Does your paper address subitem 11a-i? *

Copy and paste relevant sections from the manuscript (include quotes in quotation marks "like this" to indicate direct quotes from your manuscript), or elaborate on this item by providing additional information not in the ms, or briefly explain why the item is not applicable/relevant for your study

"Participants were then randomly assigned to the intervention or control condition using the automated simple randomization tool embedded within Qualtrics. Thus, the research team were blind to treatment allocation. However, due to the intervention's nature, actual treatment assignment was not concealed from participants."

11a-ii) Discuss e.g., whether participants knew which intervention was the “intervention of interest” and which one was the “comparator”

Informed consent procedures (4a-ii) can create biases and certain expectations - discuss e.g., whether participants knew which intervention was the “intervention of interest” and which one was the “comparator”.

	1	2	3	4	5	
subitem not at all important	○	○	○	○	◉	essential

Clear selection

Does your paper address subitem 11a-ii?

Copy and paste relevant sections from the manuscript (include quotes in quotation marks "like this" to indicate direct quotes from your manuscript), or elaborate on this item by providing additional information not in the ms, or briefly explain why the item is not applicable/relevant for your study

"Participants were then randomly assigned to the intervention or control condition using the automated simple randomization tool embedded within Qualtrics. Thus, the research team were blind to treatment allocation. However, due to the intervention's nature, actual treatment assignment was not concealed from participants."

11b) If relevant, description of the similarity of interventions

(this item is usually not relevant for ehealth trials as it refers to similarity of a placebo or sham intervention to a active medication/intervention)

Does your paper address CONSORT subitem 11b? *

Copy and paste relevant sections from the manuscript (include quotes in quotation marks "like this" to indicate direct quotes from your manuscript), or elaborate on this item by providing additional information not in the ms, or briefly explain why the item is not applicable/relevant for your study

"Participants allocated to the attention control group were asked to complete five additional measures, including a Symptom Importance Rating Questionnaire, the Chalder Fatigue Questionnaire [48], the Pittsburgh Sleep Quality Index [49], the Snaith-Hamilton Pleasure Scale [50], and the Fatigue Associated with Depression Scale [51]. These measures are not reported in this study. These were not completed by the intervention group nor reported as main outcomes on the RCT and will be reported elsewhere."

12a) Statistical methods used to compare groups for primary and secondary outcomes

NPT: When applicable, details of whether and how the clustering by care providers or centers was addressed

Does your paper address CONSORT subitem 12a? *

Copy and paste relevant sections from the manuscript (include quotes in quotation marks "like this" to indicate direct quotes from your manuscript), or elaborate on this item by providing additional information not in the ms, or briefly explain why the item is not applicable/relevant for your study

"All quantitative data were analyzed descriptively overall and by each arm. Continuous data was summarized using means (M) and standard deviations (SD). Categorical data were presented using frequencies and percentages. Data on feasibility (i.e., recruitment, intervention engagement and outcome completion rates) and acceptability (i.e., intervention acceptability and appropriateness) was reported along with baseline characteristics in the two trial arms. All outcome data were analyzed using linear mixed models (LMMs), adjusted for individual-level variation in baseline measures.
To address the first research question, intervention and control groups were compared based on complete case data at (i) the 2-week follow-up, (ii) 4-week follow-up, and 2-week and 4-week follow-up data combined using intention to treat (ITT). Between-group differences are presented as adjusted mean differences and 95% confidence intervals (CIs). Effect sizes were also calculated for the results of this study. For the second research question, exploratory moderation analyses were conducted to determine if baseline characteristics moderated the relationship between group assignment (Intervention or Control group) and each outcome measure. To address the third research question, ratings of acceptability and perceived utility (at post-intervention) were summarized using means and standard deviations. Sensitivity analysis using multiple imputation was conducted to assess the likely impact of missing data. Data were first analyzed using intention to treat (ITT) (i.e., all participants randomized who provided follow-up data). The data was then analyzed using ITT with all imputed data."

12a-i) Imputation techniques to deal with attrition / missing values

Imputation techniques to deal with attrition / missing values: Not all participants will use the intervention/comparator as intended and attrition is typically high in ehealth trials. Specify how participants who did not use the application or dropped out from the trial were treated in the statistical analysis (a complete case analysis is strongly discouraged, and simple imputation techniques such as LOCF may also be problematic [4]).

	1	2	3	4	5	
subitem not at all important	○	○	○	○	●	essential

Clear selection

Does your paper address subitem 12a-i? *

Copy and paste relevant sections from the manuscript (include quotes in quotation marks "like this" to indicate direct quotes from your manuscript), or elaborate on this item by providing additional information not in the ms, or briefly explain why the item is not applicable/relevant for your study

"Sensitivity analysis using multiple imputation was conducted to assess the likely impact of missing data. Data were first analyzed using intention to treat (ITT) (i.e., all participants randomized who provided follow-up data). The data was then analyzed using ITT with all imputed data."

12b) Methods for additional analyses, such as subgroup analyses and adjusted analyses

Does your paper address CONSORT subitem 12b? *

Copy and paste relevant sections from the manuscript (include quotes in quotation marks "like this" to indicate direct quotes from your manuscript), or elaborate on this item by providing additional information not in the ms, or briefly explain why the item is not applicable/relevant for your study

"Effect sizes were also calculated for the results of this study. For the second research question, exploratory moderation analyses were conducted to determine if baseline characteristics moderated the relationship between group assignment (Intervention or Control group) and each outcome measure. To address the third research question, ratings of acceptability and perceived utility (at post-intervention) were summarized using means and standard deviations. "

X26) REB/IRB Approval and Ethical Considerations [recommended as subheading under "Methods"] (not a CONSORT item)

X26-i) Comment on ethics committee approval

	1	2	3	4	5	
subitem not at all important	○	○	○	○	◉	essential

Clear selection

Does your paper address subitem X26-i?

Copy and paste relevant sections from the manuscript (include quotes in quotation marks "like this" to indicate direct quotes from your manuscript), or elaborate on this item by providing additional information not in the ms, or briefly explain why the item is not applicable/relevant for your study

"Ethical approval was granted by the University of Bath Psychology Research Ethics Committee (Ref: 21-212). Reciprocity was also granted by the University of Reading and Kings College London."

x26-ii) Outline informed consent procedures

Outline informed consent procedures e.g., if consent was obtained offline or online (how? Checkbox, etc.?), and what information was provided (see 4a-ii). See [6] for some items to be included in informed consent documents.

	1	2	3	4	5	
subitem not at all important	○	○	○	○	◉	essential

Clear selection

Does your paper address subitem X26-ii?

Copy and paste relevant sections from the manuscript (include quotes in quotation marks "like this" to indicate direct quotes from your manuscript), or elaborate on this item by providing additional information not in the ms, or briefly explain why the item is not applicable/relevant for your study

"Interested students were directed to a web based Qualtrics survey including an information sheet, consent form baseline assessment survey, experimental conditions, post-treatment survey, and debrief sheet. The information sheet explained the purposes of the research and the process of data collection and management. Following completion of the consent form, participants were directed to complete a baseline assessment survey measuring participants' mental health and wellbeing."

X26-iii) Safety and security procedures

Safety and security procedures, incl. privacy considerations, and any steps taken to reduce the likelihood or detection of harm (e.g., education and training, availability of a hotline)

	1	2	3	4	5	
subitem not at all important	○	○	○	○	◉	essential

Clear selection

Does your paper address subitem X26-iii?

Copy and paste relevant sections from the manuscript (include quotes in quotation marks "like this" to indicate direct quotes from your manuscript), or elaborate on this item by providing additional information not in the ms, or briefly explain why the item is not applicable/relevant for your study

"Due to the pseudo-anonymous nature of the study, distress management was based on signposting, without direct or personal contact from the research team. It was the responsibility of the participant to decide whether they acted on this advice. If a participant scored > 0 on the PHQ-9 item which asks about suicidal ideation (item 9), the participant saw an additional pop-up box suggesting that they may want to seek extra help, with a list of potential sources and contact details. They were reminded that the research team will not routinely monitor the answers to these questions. However, these participants were still included in the study."

RESULTS

13a) For each group, the numbers of participants who were randomly assigned, received intended treatment, and were analysed for the primary outcome
NPT: The number of care providers or centers performing the intervention in each group and the number of patients treated by each care provider in each center

Does your paper address CONSORT subitem 13a? *

Copy and paste relevant sections from the manuscript (include quotes in quotation marks "like this" to indicate direct quotes from your manuscript), or elaborate on this item by providing additional information not in the ms, or briefly explain why the item is not applicable/relevant for your study

"468 completed baseline measures and were randomized to either the COMET intervention (n=239) or the attention control (n=229)."

13b) For each group, losses and exclusions after randomisation, together with reasons

Does your paper address CONSORT subitem 13b? (NOTE: Preferably, this is shown in a CONSORT flow diagram) *

Copy and paste relevant sections from the manuscript (include quotes in quotation marks "like this" to indicate direct quotes from your manuscript), or elaborate on this item by providing additional information not in the ms, or briefly explain why the item is not applicable/relevant for your study

"212 participants completed COMET (88.70%), with 203 completing the post-treatment survey, and 213 individuals completed the attention control (93.01%), with 204 completing the post-attention control survey. Of those randomized, 147 participants completed the 2-week follow-up survey, 118 completed the 4-week follow-up survey, and 89 participants completed both follow-up surveys. "

13b-i) Attrition diagram

Strongly recommended: An attrition diagram (e.g., proportion of participants still logging in or using the intervention/comparator in each group plotted over time, similar to a survival curve) or other figures or tables demonstrating usage/dose/engagement.

	1	2	3	4	5	
subitem not at all important	○	○	○	○	◉	essential

Clear selection

Does your paper address subitem 13b-i?

Copy and paste relevant sections from the manuscript or cite the figure number if applicable (include quotes in quotation marks "like this" to indicate direct quotes from your manuscript), or elaborate on this item by providing additional information not in the ms, or briefly explain why the item is not applicable/relevant for your study

Figure 1. CONSORT shows attrition

14a) Dates defining the periods of recruitment and follow-up

Does your paper address CONSORT subitem 14a? *

Copy and paste relevant sections from the manuscript (include quotes in quotation marks "like this" to indicate direct quotes from your manuscript), or elaborate on this item by providing additional information not in the ms, or briefly explain why the item is not applicable/relevant for your study

Figure 1. CONSORT shows recruitment and follow-up

14a-i) Indicate if critical "secular events" fell into the study period

Indicate if critical "secular events" fell into the study period, e.g., significant changes in Internet resources available or "changes in computer hardware or Internet delivery resources"

	1	2	3	4	5	
subitem not at all important	○	○	○	○	◉	essential

Clear selection

Does your paper address subitem 14a-i?

Copy and paste relevant sections from the manuscript (include quotes in quotation marks "like this" to indicate direct quotes from your manuscript), or elaborate on this item by providing additional information not in the ms, or briefly explain why the item is not applicable/relevant for your study

"Participant recruitment and follow-up took place between September 2021 and December 2022."

14b) Why the trial ended or was stopped (early)

Does your paper address CONSORT subitem 14b? *

Copy and paste relevant sections from the manuscript (include quotes in quotation marks "like this" to indicate direct quotes from your manuscript), or elaborate on this item by providing additional information not in the ms, or briefly explain why the item is not applicable/relevant for your study

Not applicable as trial not stopped early

15) A table showing baseline demographic and clinical characteristics for each group

NPT: When applicable, a description of care providers (case volume, qualification, expertise, etc.) and centers (volume) in each group

Does your paper address CONSORT subitem 15? *

Copy and paste relevant sections from the manuscript (include quotes in quotation marks "like this" to indicate direct quotes from your manuscript), or elaborate on this item by providing additional information not in the ms, or briefly explain why the item is not applicable/relevant for your study

Please see table 1 for baseline

15-i) Report demographics associated with digital divide issues

In ehealth trials it is particularly important to report demographics associated with digital divide issues, such as age, education, gender, social-economic status, computer/Internet/ehealth literacy of the participants, if known.

	1	2	3	4	5	
subitem not at all important	○	○	◉	○	○	essential

Clear selection

Does your paper address subitem 15-i? *

Copy and paste relevant sections from the manuscript (include quotes in quotation marks "like this" to indicate direct quotes from your manuscript), or elaborate on this item by providing additional information not in the ms, or briefly explain why the item is not applicable/relevant for your study

Please see table 1 for baseline

16) For each group, number of participants (denominator) included in each analysis and whether the analysis was by original assigned groups

16-i) Report multiple “denominators” and provide definitions

Report multiple “denominators” and provide definitions: Report N’s (and effect sizes) “across a range of study participation [and use] thresholds” [1], e.g., N exposed, N consented, N used more than x times, N used more than y weeks, N participants “used” the intervention/comparator at specific pre-defined time points of interest (in absolute and relative numbers per group). Always clearly define “use” of the intervention.

	1	2	3	4	5	
subitem not at all important	○	○	○	○	◉	essential

Clear selection

Does your paper address subitem 16-i? *

Copy and paste relevant sections from the manuscript (include quotes in quotation marks "like this" to indicate direct quotes from your manuscript), or elaborate on this item by providing additional information not in the ms, or briefly explain why the item is not applicable/relevant for your study

Please see table 1 for baseline

16-ii) Primary analysis should be intent-to-treat

Primary analysis should be intent-to-treat, secondary analyses could include comparing only “users”, with the appropriate caveats that this is no longer a randomized sample (see 18-i).

	1	2	3	4	5	
subitem not at all important	○	○	○	○	◉	essential

Clear selection

Does your paper address subitem 16-ii?

Copy and paste relevant sections from the manuscript (include quotes in quotation marks "like this" to indicate direct quotes from your manuscript), or elaborate on this item by providing additional information not in the ms, or briefly explain why the item is not applicable/relevant for your study

"For the complete-case, ITT, analysis, at the 2-week follow-up, subjective wellbeing (WEMWBS) – was significantly higher in the intervention group compared with the control, with a mean difference (MD) of 1.39 (95% CI 0.19 to 2.61, p=.026)."

17a) For each primary and secondary outcome, results for each group, and the estimated effect size and its precision (such as 95% confidence interval)

Does your paper address CONSORT subitem 17a? *

Copy and paste relevant sections from the manuscript (include quotes in quotation marks "like this" to indicate direct quotes from your manuscript), or elaborate on this item by providing additional information not in the ms, or briefly explain why the item is not applicable/relevant for your study

"For the complete-case, ITT, analysis, at the 2-week follow-up, subjective wellbeing (WEMWBS) – was significantly higher in the intervention group compared with the control, with a mean difference (MD) of 1.39 (95% CI 0.19 to 2.61, p=.026). Depression was significantly lower in the intervention group compared with the control at the 2-week (PHQ-9; MD = -1.31, 95% CI -2.51 to -0.12, p=.033) and combined 2- and 4-week follow-up (PHQ-9; MD = -0.86, 95% CI -1.71 to -0.02, p=.047).
Perceived stress was significantly lower in the intervention group compared with the control at the 2-week follow-up (PSS-4; MD = -1.33, 95% CI -2.10 to -0.57, p<.001). For the remaining outcome measures (i.e., GAD-7, NAS, and PAS), no between-group differences were observed at 2-week or 4-week follow-ups (Table 2)."

17a-i) Presentation of process outcomes such as metrics of use and intensity of use

In addition to primary/secondary (clinical) outcomes, the presentation of process outcomes such as metrics of use and intensity of use (dose, exposure) and their operational definitions is critical. This does not only refer to metrics of attrition (13-b) (often a binary variable), but also to more continuous exposure metrics such as "average session length". These must be accompanied by a technical description how a metric like a "session" is defined (e.g., timeout after idle time) [1] (report under item 6a).

	1	2	3	4	5	
subitem not at all important	○	○	●	○	○	essential

Clear selection

Does your paper address subitem 17a-i?

Copy and paste relevant sections from the manuscript (include quotes in quotation marks "like this" to indicate direct quotes from your manuscript), or elaborate on this item by providing additional information not in the ms, or briefly explain why the item is not applicable/relevant for your study

"Overall, participants found COMET to be acceptable with between 82% and 93% of participants agreeing or strongly agreeing that they approved of, liked and welcomed COMET and found it appealing. Participants also found COMET to be appropriate with 80% to 93% agreeing or strongly agreeing that COMET was fitting, suitable, applicable and a good match (Table 3). Each of COMET's four modules was also perceived to have high utility (Table 4). Most participants at least slightly agreed that behavioral activation (83% - 89%), cognitive restructuring (71%-86%), gratitude (86%-90%) and self-compassion (74%-86%) were helpful, engaging, and applicable. However, participants generally liked the self-compassion module the most and the behavioral activation module the least (Figure 1)."

17b) For binary outcomes, presentation of both absolute and relative effect sizes is recommended

Does your paper address CONSORT subitem 17b? *

Copy and paste relevant sections from the manuscript (include quotes in quotation marks "like this" to indicate direct quotes from your manuscript), or elaborate on this item by providing additional information not in the ms, or briefly explain why the item is not applicable/relevant for your study

No binary outcomes used in this paper

18) Results of any other analyses performed, including subgroup analyses and adjusted analyses, distinguishing pre-specified from exploratory

Does your paper address CONSORT subitem 18? *

Copy and paste relevant sections from the manuscript (include quotes in quotation marks "like this" to indicate direct quotes from your manuscript), or elaborate on this item by providing additional information not in the ms, or briefly explain why the item is not applicable/relevant for your study

"Intervention outcomes - Moderation analysis
For the complete case analyses, the interaction between group allocation and receiving current treatment on subjective wellbeing at 2-week (B = -3.37, 95% CI -6.34 to -0.41, p=.028), 4 week (B = -4.11, 95% CI -7.57 to -0.65, p=.023) and 2 and 4 week follow up (B = -2.00, 95% CI -3.97 to -0.02, p=.005) was significant and negative. The interaction between group allocation and age was significant and positive at 4-week for depression (B = 0.19, 95% CI 0.01 to 0.37, p=.040) and anxiety (B = 0.15, 95% CI 0.01 to 0.30, p=.045). The interaction between group allocation and previous diagnosis was significant and positive for depression at 2 and 4 weeks (B = 1.94, 95% CI 0.13 to 3.76, p=.038). The interaction between group allocation and baseline depression was significant and negative for depression 4-weeks (B = -0.22, 95% CI -0.44 to 0.00, p=.055) and significant and negative for perceived stress at 2 and 4 weeks (B = -0.11, 95% CI -0.20 to -0.01, p=.038). Finally, the interaction between group allocation and baseline anxiety was significant and negative for perceived stress at 4 weeks (B = -0.23, 95% CI -0.45 to -0.01, p=.047). No other interaction effects were observed. See Multimedia Appendix 2 for full analysis.

Intervention outcomes - Sensitivity analyses
Missingness ranged from 0.00 to 68.42% for cases (M = 44.46%, SD = 25.28%) and from 0.00 to 73.29% (M = 44.46%, SD = 34.93%) for variables. Little's MCAR test was applied and indicated that the data were missing completely at random ($\chi^2(100) = 99.10$, p = .508); hence, it was assumed that missingness was purely random and not related to any observed or unobserved data.
Given the high proportion of missing data, multiple imputation was carried out to estimate follow-up outcomes for all participants who did not provide data at 2-week and 4-week follow-ups (Table S1, Multimedia Appendix 3). Depression and perceived stress were lower in the intervention group compared with the control at the 2-week follow-up but only bordering significance (PHQ-9; MD = -0.53, 95% CI -1.10 to 0.04, p=.068 and PSS-4; MD = -0.57, 95% CI -1.15 to 0.01, p=.054). No between-group effects between the intervention and control were for any of the other variables or follow-up points."

18-i) Subgroup analysis of comparing only users

A subgroup analysis of comparing only users is not uncommon in ehealth trials, but if done, it must be stressed that this is a self-selected sample and no longer an unbiased sample from a randomized trial (see 16-iii).

	1	2	3	4	5	
subitem not at all important	(selected)	○	○	○	○	essential

Clear selection

Does your paper address subitem 18-i?

Copy and paste relevant sections from the manuscript (include quotes in quotation marks "like this" to indicate direct quotes from your manuscript), or elaborate on this item by providing additional information not in the ms, or briefly explain why the item is not applicable/relevant for your study

Your answer

19) All important harms or unintended effects in each group
(for specific guidance see CONSORT for harms)

Does your paper address CONSORT subitem 19? *

Copy and paste relevant sections from the manuscript (include quotes in quotation marks "like this" to indicate direct quotes from your manuscript), or elaborate on this item by providing additional information not in the ms, or briefly explain why the item is not applicable/relevant for your study

No harms reported in trial

19-i) Include privacy breaches, technical problems

Include privacy breaches, technical problems. This does not only include physical "harm" to participants, but also incidents such as perceived or real privacy breaches [1], technical problems, and other unexpected/unintended incidents. "Unintended effects" also includes unintended positive effects [2].

	1	2	3	4	5	
subitem not at all important	(•)	( )	( )	( )	( )	essential

Clear selection

Does your paper address subitem 19-i?

Copy and paste relevant sections from the manuscript (include quotes in quotation marks "like this" to indicate direct quotes from your manuscript), or elaborate on this item by providing additional information not in the ms, or briefly explain why the item is not applicable/relevant for your study

Your answer

19-ii) Include qualitative feedback from participants or observations from staff/researchers

Include qualitative feedback from participants or observations from staff/researchers, if available, on strengths and shortcomings of the application, especially if they point to unintended/unexpected effects or uses. This includes (if available) reasons for why people did or did not use the application as intended by the developers.

	1	2	3	4	5	
subitem not at all important	( )	( )	(•)	( )	( )	essential

Clear selection

Does your paper address subitem 19-ii?

Copy and paste relevant sections from the manuscript (include quotes in quotation marks "like this" to indicate direct quotes from your manuscript), or elaborate on this item by providing additional information not in the ms, or briefly explain why the item is not applicable/relevant for your study

"Overall, participants found COMET to be acceptable with between 82% and 93% of participants agreeing or strongly agreeing that they approved of, liked and welcomed COMET and found it appealing. Participants also found COMET to be appropriate with 80% to 93% agreeing or strongly agreeing that COMET was fitting, suitable, applicable and a good match (Table 3). Each of COMET's four modules was also perceived to have high utility (Table 4). Most participants at least slightly agreed that behavioral activation (83% - 89%), cognitive restructuring (71%-86%), gratitude (86%-90%) and self-compassion (74%-86%) were helpful, engaging, and applicable. However, participants generally liked the self-compassion module the most and the behavioral activation module the least (Figure 1)."

DISCUSSION

22) Interpretation consistent with results, balancing benefits and harms, and considering other relevant evidence

NPT: In addition, take into account the choice of the comparator, lack of or partial blinding, and unequal expertise of care providers or centers in each group

22-i) Restate study questions and summarize the answers suggested by the data, starting with primary outcomes and process outcomes (use)

Restate study questions and summarize the answers suggested by the data, starting with primary outcomes and process outcomes (use).

	1	2	3	4	5	
subitem not at all important	○	○	○	○	◉	essential

Clear selection

Does your paper address subitem 22-i? *

Copy and paste relevant sections from the manuscript (include quotes in quotation marks "like this" to indicate direct quotes from your manuscript), or elaborate on this item by providing additional information not in the ms, or briefly explain why the item is not applicable/relevant for your study

"UK university students engaged well with the COMET online SSI and exhibited small, significant improvements in wellbeing, depression severity, and perceived stress over a 2-week follow-up period compared to the Control Arm. Changes in anxiety severity, positive affect, and negative affect were nonsignificant. Exploratory analysis also revealed that COMET was potentially more effective at reducing stress for those with elevated symptoms of depression or anxiety. We also found that COMET-GB, was largely well-received in terms of acceptability, appropriateness, and feasibility, although users commented that it was too long, and some had technical issues."

22-ii) Highlight unanswered new questions, suggest future research

Highlight unanswered new questions, suggest future research.

	1	2	3	4	5	
subitem not at all important	○	○	○	○	●	essential

Clear selection

Does your paper address subitem 22-ii?

Copy and paste relevant sections from the manuscript (include quotes in quotation marks "like this" to indicate direct quotes from your manuscript), or elaborate on this item by providing additional information not in the ms, or briefly explain why the item is not applicable/relevant for your study

"Future studies should further explore how best to support underserved groups (e.g., explore experiences of minority groups [75]). AI (Artificial Intelligence) driven adaptive trials may also help us to answer what works for whom [76]. To reach university students before mental health symptoms become functionally impairing, early interventions or prevention may have better reach by embedding them within courses and maximal engagement if coproduced [77]."

20) Trial limitations, addressing sources of potential bias, imprecision, and, if relevant, multiplicity of analyses

20-i) Typical limitations in ehealth trials

Typical limitations in ehealth trials: Participants in ehealth trials are rarely blinded. Ehealth trials often look at a multiplicity of outcomes, increasing risk for a Type I error. Discuss biases due to non-use of the intervention/usability issues, biases through informed consent procedures, unexpected events.

	1	2	3	4	5	
subitem not at all important	○	○	○	○	●	essential

Clear selection

Does your paper address subitem 20-i? *

Copy and paste relevant sections from the manuscript (include quotes in quotation marks "like this" to indicate direct quotes from your manuscript), or elaborate on this item by providing additional information not in the ms, or briefly explain why the item is not applicable/relevant for your study

"We evaluated an existing intervention in a novel population, using a broad, well-validated series of psychometric instruments which spanned different dimensions of mental health problems and wellbeing. This is important, given that no single measure captures all stakeholder priorities in university student mental health [71] and our comprehensive approach including both mental health symptom measures and a wellbeing measure allowed for a more holistic understanding of COMET's impact.
However, like other digital mental health intervention studies in university student samples [63-67], we had high attrition rates (57%). Although the absence of patterned missingness suggests that this attrition did not bias the results, it nonetheless substantially impacted the sample size. Thus, although we recruited and randomized 425 participants, only 193 were retained at follow-up. Our a priori calculations indicated that a sample size of 378 participants would be required to detect statistically significant differences in the PHQ-9. Therefore, we had insufficient power and findings may subsequently be prone to Type II errors.
Most participants were young, white, heterosexual women, like other university mental health intervention studies [62, 72]. While this helps to provide valuable insights into the effects of COMET within this demographic, it does pose a limitation regarding the generalizability of the findings to the broader spectrum of UK university students. Furthermore, due to insufficient diversity in the sample, exploratory moderation analyses for demographic variables could not be meaningfully conducted.
While the efficacy of COMET was established across various domains, it is important to note that contrary to real-world settings, participants were given either monetary rewards or course credit incentives for completing these interventions. This raises concerns about the applicability and genuine impact of COMET outside of an incentivized research context [73]. Accordingly, to ensure results are driven by inherent value and user commitment rather than external rewards, future research should examine the intervention's impact in contexts devoid of external motivators.
The current study similarly focused on short-term 2-week and 4-week outcomes, leaving questions about sustained efficacy across the longer term. Future investigations should emphasize extended follow-ups to provide a comprehensive understanding of an intervention's enduring benefits [74]. In contemporary psychotherapeutic research, a benchmark of at least six months is considered standard."

21) Generalisability (external validity, applicability) of the trial findings

NPT: External validity of the trial findings according to the intervention, comparators, patients, and care providers or centers involved in the trial

21-i) Generalizability to other populations

Generalizability to other populations: In particular, discuss generalizability to a general Internet population, outside of a RCT setting, and general patient population, including applicability of the study results for other organizations

	1	2	3	4	5	
subitem not at all important	○	○	●	○	○	essential

Clear selection

Does your paper address subitem 21-i?

Copy and paste relevant sections from the manuscript (include quotes in quotation marks "like this" to indicate direct quotes from your manuscript), or elaborate on this item by providing additional information not in the ms, or briefly explain why the item is not applicable/relevant for your study

"This study demonstrated the preliminary short-term effectiveness of the COMET intervention, as evidenced by the significant between-group differences favoring the intervention at the 2-week follow-up. However, attrition was high, potentially biasing the results. Participant feedback indicated overall satisfaction with the intervention, with perceived accessibility, immediate benefits, and potential long-term impact being notable findings. These findings support the potential value of COMET as a mental health intervention and highlight important areas for further development in future SSI interventions."

21-ii) Discuss if there were elements in the RCT that would be different in a routine application setting

Discuss if there were elements in the RCT that would be different in a routine application setting (e.g., prompts/reminders, more human involvement, training sessions or other co-interventions) and what impact the omission of these elements could have on use, adoption, or outcomes if the intervention is applied outside of a RCT setting.

	1	2	3	4	5	
subitem not at all important	○	●	○	○	○	essential

Clear selection

Does your paper address subitem 21-ii?

Copy and paste relevant sections from the manuscript (include quotes in quotation marks "like this" to indicate direct quotes from your manuscript), or elaborate on this item by providing additional information not in the ms, or briefly explain why the item is not applicable/relevant for your study

Your answer

OTHER INFORMATION

23) Registration number and name of trial registry

Does your paper address CONSORT subitem 23? *

Copy and paste relevant sections from the manuscript (include quotes in quotation marks "like this" to indicate direct quotes from your manuscript), or elaborate on this item by providing additional information not in the ms, or briefly explain why the item is not applicable/relevant for your study

ClinicalTrials.gov NCT05718141

24) Where the full trial protocol can be accessed, if available

Does your paper address CONSORT subitem 24? *

Cite a Multimedia Appendix, other reference, or copy and paste relevant sections from the manuscript (include quotes in quotation marks "like this" to indicate direct quotes from your manuscript), or elaborate on this item by providing additional information not in the ms, or briefly explain why the item is not applicable/relevant for your study

ClinicalTrials.gov NCT05718141

25) Sources of funding and other support (such as supply of drugs), role of funders

Does your paper address CONSORT subitem 25? *

Copy and paste relevant sections from the manuscript (include quotes in quotation marks "like this" to indicate direct quotes from your manuscript), or elaborate on this item by providing additional information not in the ms, or briefly explain why the item is not applicable/relevant for your study

No other sources of funding and other support

X27) Conflicts of Interest (not a CONSORT item)

X27-i) State the relation of the study team towards the system being evaluated

In addition to the usual declaration of interests (financial or otherwise), also state the relation of the study team towards the system being evaluated, i.e., state if the authors/evaluators are distinct from or identical with the developers/sponsors of the intervention.

	1	2	3	4	5	
subitem not at all important	(•)	( )	( )	( )	( )	essential

Clear selection

Does your paper address subitem X27-i?

Copy and paste relevant sections from the manuscript (include quotes in quotation marks "like this" to indicate direct quotes from your manuscript), or elaborate on this item by providing additional information not in the ms, or briefly explain why the item is not applicable/relevant for your study

Your answer

About the CONSORT EHEALTH checklist

As a result of using this checklist, did you make changes in your manuscript? *

- ○ yes, major changes
- ○ yes, minor changes
- ◉ no

What were the most important changes you made as a result of using this checklist?

Your answer

How much time did you spend on going through the checklist INCLUDING making changes in your manuscript *

Around an hour was spent

As a result of using this checklist, do you think your manuscript has improved? *

- ○ yes
- ◉ no
- ○ Other:

Would you like to become involved in the CONSORT EHEALTH group?

This would involve for example becoming involved in participating in a workshop and writing an "Explanation and Elaboration" document

- (x) yes
- ( ) no
- ( ) Other:

Clear selection

Any other comments or questions on CONSORT EHEALTH

The process seems a little rigid and reputative. Would be better signposting to the manuscript rather than copying and pasting text. The section on importance seems a little perfunctory.

STOP - Save this form as PDF before you click submit

To generate a record that you filled in this form, we recommend to generate a PDF of this page (on a Mac, simply select "print" and then select "print as PDF") before you submit it.

When you submit your (revised) paper to JMIR, please upload the PDF as supplementary file.

Don't worry if some text in the textboxes is cut off, as we still have the complete information in our database. Thank you!

Final step: Click submit !

Click submit so we have your answers in our database!

Submit Clear form

Never submit passwords through Google Forms.

This content is neither created nor endorsed by Google. Report Abuse - Terms of Service - Privacy Policy

Google Forms
