# Supplementary material for: Investigating the Efficacy of the Web-Based Common Elements Toolbox (COMET) Single-Session Interventions in Improving UK University Student Well-Being: Randomized Controlled Trial
Source: J Med Internet Res. 2025 Jan 31;27:e58164. doi: 10.2196/58164 (PMC11829182; doi:10.2196/58164)
Supplement: Multimedia Appendix 2 [file jmir_v27i1e58164_app2.docx]

Table S1. Interaction between group allocation and age

| Outcome | Between-group difference, 95% CI | *P*-value |
| --- | --- | --- |
| WEMWBS |  |  |
| 2-weeks | 0.03 (-0.17 to 0.22) | .789 |
| 4-weeks | -0.08 (-25 to 0.09) | .362 |
| 2- and 4- weeks | 0.06 (-0.06 to 0.18) | .357 |
| PHQ-9 |  |  |
| 2-weeks | -0.12 (-0.31 to 0.07) | .230 |
| 4-weeks | 0.19 (0.01 to 0.37) | .040 |
| 2- and 4- weeks | 0.0002 (-0.12 to 0.12) | .997 |
| GAD-7 |  |  |
| 2-weeks | -.004 (-0.17 to 0.16) | 0.957 |
| 4-weeks | 0.15 (0.01 to 0.30) | .045* |
| 2- and 4- weeks | .04 (-0.05 to 0.14) | .385 |
| NAS |  |  |
| 2-weeks | 0.04 (-0.26 to 0.35) | .776 |
| 4-weeks | 0.18 (-0.11 to 0.47) | .234 |
| 2- and 4- weeks | -0.01 (-0.19 to 0.18) | .937 |
| PAS |  |  |
| 2-weeks | 0.16 (-0.18 to 0.49) | .364 |
| 4-weeks | -0.15 (-0.46 to 0.15) | .335 |
| 2- and 4- weeks | 0.08 (-0.12 to 0.29) | .417 |
| PSS-4 |  |  |
| 2-weeks | -0.06 (-0.18 to 0.07) | .379 |
| 4-weeks | 0.09 (-0.03 to 0.22) | .142 |
| 2- and 4- weeks | 0.01 (-0.07 to 0.09) | .795 |

Table S2. Interaction between group allocation and gender

| Outcome | Between-group difference, 95% CI | *P*-value |
| --- | --- | --- |
| WEMWBS |  |  |
| 2-weeks | -1.71 (-5.50 to 2.07) | .380 |
| 4-weeks | -0.20 (-3.79 to 3.93) | .914 |
| 2- and 4- weeks | -0.60 (-2.80 to 1.60) | .597 |
| PHQ-9 |  |  |
| 2-weeks | 0.89 (-2.85 to 4.63) | .644 |
| 4-weeks | -0.35 (-4.30 to 3.61) | .865 |
| 2- and 4- weeks | 1.29 (-0.99 to 3.56) | .271 |
| GAD-7 |  |  |
| 2-weeks | -0.70 (-3.75 to 2.36) | .659 |
| 4-weeks | -0.11 (-3.21 to 2.99) | .943 |
| 2- and 4- weeks | 0.32 (-1.49 to 2.15) | .725 |
| NAS |  |  |
| 2-weeks | 0.62 (-5.01 to 6.24) | .831 |
| 4-weeks | -2.67 (-8.57 to 3.24) | .383 |
| 2- and 4- weeks | -1.97 (-5.35 to 1.41) | .259 |
| PAS |  |  |
| 2-weeks | 3.04 (-3.24 to 9.33) | .348 |
| 4-weeks | -0.07 (-0.45 to 0.30) | .712 |
| 2- and 4- weeks | -0.83 (-4.64 to 2.96) | .670 |
| PSS-4 |  |  |
| 2-weeks | 0.12 (-2.25 to 2.51) | .916 |
| 4-weeks | -1.10 (-3.66 to 1.47) | .410 |
| 2- and 4- weeks | -0.68 (-2.17 to 0.81) | .377 |

Table S3. Interaction between group allocation and previous diagnosis

| Outcome | Between-group difference, 95% CI | *P*-value |
| --- | --- | --- |
| WEMWBS |  |  |
| 2-weeks | -1.50 (-4.12 to 1.13) | .269 |
| 4-weeks | 0.34 (-2.86 to 3.53) | .838 |
| 2- and 4- weeks | 0.09 (-1.69 to 1.87) | .919 |
| PHQ-9 |  |  |
| 2-weeks | 2.54 (-0.03 to 5.11) | .056 |
| 4-weeks | 3.33 (-0.11 to 6.78) | .063 |
| 2- and 4- weeks | 1.94 (0.13 to 3.76) | .038 |
| GAD-7 |  |  |
| 2-weeks | 0.31 (-1.81 to 2.43) | .776 |
| 4-weeks | 0.95 (-1.78 to 3.69) | .676 |
| 2- and 4- weeks | 0.73 (-0.73 to 2.20) | .330 |
| NAS |  |  |
| 2-weeks | 1.55 (-2.44 to 5.54) | .450 |
| 4-weeks | -0.41 (-5.71 to 4.90) | .882 |
| 2- and 4- weeks | 0.10 (-2.66 to 2.86) | .945 |
| PAS |  |  |
| 2-weeks | -1.97 (-6.38 to 2.43) | .385 |
| 4-weeks | -5.30 (-10.96 to 0.35) | .071 |
| 2- and 4- weeks | -2.61 (-5.66 to 0.44) | .097 |
| PSS-4 |  |  |
| 2-weeks | 0.11 (-1.55 to 1.77) | .898 |
| 4-weeks | -0.58 (-2.87 to 1.70) | .620 |
| 2- and 4- weeks | 0.01 (-1.19 to 1.20) | .989 |

Table S4. Interaction between group allocation and current treatment

| Outcome | Between-group difference, 95% CI | *P*-value |
| --- | --- | --- |
| WEMWBS |  |  |
| 2-weeks | -3.37 (-6.34 to -0.41) | .028 |
| 4-weeks | -4.11 (-7.57 to -0.65) | .023 |
| 2- and 4- weeks | -2.00 (-3.97 to -0.02) | .050 |
| PHQ-9 |  |  |
| 2-weeks | 0.42 (-2.55 to 3.39) | .783 |
| 4-weeks | 2.88 (-0.92 to 6.68) | .144 |
| 2- and 4- weeks | 0.51 (-1.55 to 2.57) | .632 |
| GAD-7 |  |  |
| 2-weeks | 0.49 (-1.93 to 2.91) | .396 |
| 4-weeks | 2.01 (-0.95 to 4.97) | .190 |
| 2- and 4- weeks | 0.87 (-0.76 to 2.50) | .299 |
| NAS |  |  |
| 2-weeks | 0.49 (-4.06 to 5.04) | .209 |
| 4-weeks | 5.10 (-0.72 to 10.92) | .091 |
| 2- and 4- weeks | 2.83 (-0.23 to 5.90) | .073 |
| PAS |  |  |
| 2-weeks | -3.22 (-8.19 to 1.75) | .209 |
| 4-weeks | -3.64 (-9.94 to 2.66) | .264 |
| 2- and 4- weeks | -2.53 (-5.94 to 0.87) | .149 |
| PSS-4 |  |  |
| 2-weeks | 1.57 (-1.17 to 4.30) | .270 |
| 4-weeks | 1.18 (-1.34 to 3.69) | .366 |
| 2- and 4- weeks | 0.88 (-0.46 to 2.22) | .204 |

Table S5. Interaction between group allocation and baseline depression

| Outcome | Between-group difference, 95% CI | *P*-value |
| --- | --- | --- |
| WEMWBS |  |  |
| 2-weeks | 0.10 (-0.11 to 0.31) | .338 |
| 4-weeks | 0.18 (-0.04 to 0.40) | .118 |
| 2- and 4- weeks | 0.06 (-0.08 to 0.21) | .410 |
| PHQ-9 |  |  |
| 2-weeks | -0.14 (-0.35 to 0.06) | .182 |
| 4-weeks | -0.22 (-0.44 to 0.001) | .055 |
| 2- and 4- weeks | -0.07 (-0.21 to 0.08) | .373 |
| GAD-7 |  |  |
| 2-weeks | -0.03 (-0.20 to 0.14) | .713 |
| 4-weeks | -0.08 (-0.26 to 0.11) | .438 |
| 2- and 4- weeks | -0.05 (-0.17 to 0.07) | .408 |
| NAS |  |  |
| 2-weeks | -0.20 (-0.52 to 0.11) | .211 |
| 4-weeks | -0.12 (-0.49 to 0.25) | .517 |
| 2- and 4- weeks | -0.03 (-0.25 to 0.19) | .801 |
| PAS |  |  |
| 2-weeks | 0.13 (-0.23 to 0.49) | .485 |
| 4-weeks | 0.14 (-0.26 to 0.55) | .497 |
| 2- and 4- weeks | 0.07 (-0.19 to 0.32) | .608 |
| PSS-4 |  |  |
| 2-weeks | -0.07 (-0.21 to 0.06) | .286 |
| 4-weeks | -0.15 (-0.31 to 0.01) | .071 |
| 2- and 4- weeks | -0.11 (-0.20 to -0.01) | .038 |

Table S6. Interaction between group allocation and baseline anxiety

| Outcome | Between-group difference, 95% CI | *P*-value |
| --- | --- | --- |
| WEMWBS |  |  |
| 2-weeks | 0.11 (-0.15 to 0.37) | .422 |
| 4-weeks | 0.10 (-0.20 to 0.41) | .513 |
| 2- and 4- weeks | -0.02 (-0.21 to 0.16) | .792 |
| PHQ-9 |  |  |
| 2-weeks | -0.24 (-0.51 to 0.02) | .079 |
| 4-weeks | -0.31 (-0.64 to 0.01) | .063 |
| 2- and 4- weeks | -0.14 (-0.32 to 0.05) | .152 |
| GAD-7 |  |  |
| 2-weeks | -0.14 (-0.34 to 0.06) | .167 |
| 4-weeks | -0.22 (-0.46 to 0.01) | .071 |
| 2- and 4- weeks | -0.09 (-0.23 to 0.04) | .188 |
| NAS |  |  |
| 2-weeks | -0.31 (-0.70 to 0.09) | .133 |
| 4-weeks | -0.28 (-0.78 to 0.21) | .265 |
| 2- and 4- weeks | -0.07 (-0.34 to 0.20) | .625 |
| PAS |  |  |
| 2-weeks | 0.09 (-0.37 to 0.55) | .704 |
| 4-weeks | 0.07 (-0.50 to 0.64) | .811 |
| 2- and 4- weeks | 0.003 (-0.31 to 0.32) | .988 |
| PSS-4 |  |  |
| 2-weeks | -0.10 (-0.27 to 0.07) | .278 |
| 4-weeks | -0.23 (-0.45 to -0.01) | .047 |
| 2- and 4- weeks | -0.12 (-0.24 to 0.01) | .065 |
