# Supplementary material for: Investigating the Efficacy of the Web-Based Common Elements Toolbox (COMET) Single-Session Interventions in Improving UK University Student Well-Being: Randomized Controlled Trial
Source: J Med Internet Res. 2025 Jan 31;27:e58164. doi: 10.2196/58164 (PMC11829182; doi:10.2196/58164)
Supplement: Multimedia Appendix 3 [file jmir_v27i1e58164_app3.docx]

Table S1. Sensitivity analysis

| Outcome | Pooled Between-group difference, 95% CI | *P*-value |
| --- | --- | --- |
| WEMWBS |  |  |
| 2-weeks | 0.21 (-0.74 to 1.16) | .666 |
| 4-weeks | 0.01 (-0.96 to 0.98) | .984 |
| 2- and 4- weeks | 0.004 (-0.48 to 0.49) | .986 |
| PHQ-9 |  |  |
| 2-weeks | -0.53 (-1.10 to 0.04) | .068 |
| 4-weeks | -0.45 (-1.09 to 0.18) | .163 |
| 2- and 4- weeks | -0.20 (-0.63 to 0.24) | .383 |
| GAD-7 |  |  |
| 2-weeks | -0.12 (-0.58 to 0.33) | .592 |
| 4-weeks | -0.16 (-0.66 to 0.34) | .541 |
| 2- and 4- weeks | -0.06 (-0.38 to 0.25) | .700 |
| NAS |  |  |
| 2-weeks | -0.08 (-1.53 to 1.37) | .911 |
| 4-weeks | -0.43 (-1.96 to 1.11) | .588 |
| 2- and 4- weeks | -0.21 (-0.99 to 0.57) | .596 |
| PAS |  |  |
| 2-weeks | 1.00 (-0.66 to 2.65) | .238 |
| 4-weeks | 0.19 (-1.58 to 1.96) | .834 |
| 2- and 4- weeks | 0.06 (-0.80 to 0.92) | .894 |
| PSS-4 |  |  |
| 2-weeks | -0.57 (-1.15 to 0.01) | .054 |
| 4-weeks | -0.31 (-0.92 to 0.31) | .330 |
| 2- and 4- weeks | -0.14 (-0.45 to 0.17) | .378 |
