# Supplementary material for: Investigating the Efficacy of the Web-Based Common Elements Toolbox (COMET) Single-Session Interventions in Improving UK University Student Well-Being: Randomized Controlled Trial
Source: J Med Internet Res. 2025 Jan 31;27:e58164. doi: 10.2196/58164 (PMC11829182; doi:10.2196/58164)
Supplement: Multimedia Appendix 4 [file jmir_v27i1e58164_app4.docx]

**Qualitative analysis**

The free text content analysis of participant's experiences of COMET resulted in the formulation of four categories (see Table S1).

*Time/length*

Participants expressed that some of the exercises, like the ABCD exercise (where participants were asked to identify an activating stressful/upsetting event, and consider thoughts, beliefs, alternative perspectives, and subsequent change in their own thoughts), were too long, leading to potential dropouts from the study. Some participants also mentioned feeling bored during the intervention, which again may have contributed to participant attrition.

*Accessibility*

Several participants reported technical issues (e.g., images not loading), acting as a potential barrier to engagement. However, participants felt that COMET introduced valuable skills in a manner that was simple and easy to understand, which was a positive.

*Immediate effects*

Many participants reported feeling positive, relaxed, and self-compassionate after completing COMET. They also reported that the intervention helped to improve their thinking and outlook by rationalising worries and recognising the positive aspects of their lives.

*Long-term effects*

Participants acknowledged the importance of planning positive active activities to regain a healthy routine. While they did not anticipate long-term changes in their handling of stressful situations or self-critical tendencies, COMET encouraged them to consider restructuring their thinking, which could potentially have a longer-term impact.

Table S1. Categories and example quotes from qualitative analysis.

| **Category** | **Illustrative quotes** |
| --- | --- |
| Time/length | - "...but found the ABCD exercise a bit long and think that most people would drop out when they got to that part."^a^ - "I did get a bit bored and was unsure how much longer this would be."^a^ |
| Accessibility | - "Some of the images did not load for me."^a^ - "Introduces good skills in an easy way."^b^ |
| Immediate effects | - "After doing this, I feel so positive about myself and I feel so relaxed after this self-compassion session."^c^ - "I hope you enjoy it and feel relaxed and happy, the way I am feeling now."^c^ - "Improve my thinking and outlook."^b^ - "It helped me to realise how lucky I am in life, all the wonderful things I have around me. it helped me to rationalise my worries."^b^ |
| Long-term effects | - "You can plan active activities to get your life back to a routine."^c^ - "Found that positive activities could be a useful tool to use in my routine."^b^ - "I don't think this will change the way I handle stressful situations or how self-critical I am long term, but it's been helpful to consider restructuring my thinking."^a^ - "Little things could lead to positive change."^c^ - "I'd also really have appreciated having my answers to each question sent to me, as they would be helpful for my practice in the next few weeks and the future."^a^ |

Note:

^a^ = Response to “We'd like to see if you have any feedback about things you liked or disliked about the program. If you have any feedback for us, please tell us below”.

^b^ = Response to “Why did you choose that option? In what ways do you think it could be helpful?”.

^c^ = Response to “Please write your self-compassion letter in the box below”.

Participants also provided feedback with specific suggestions for how COMET could be improved, including making the wording more empowering and natural, fixing the technical issues, making the text more concise, and abbreviating the writing exercise to a positive mantra that can be recorded on a mobile phone. Participants also wanted ways that would help them remember the information presented, for example, adding a look-up function for keywords.
